# Maternal mental disorders and neonatal outcomes: Danish population-based cohort study

**DOI:** 10.1192/bjp.2024.164

**Published:** 2025-01

**Authors:** Natalie C. Momen, Hannah Chatwin, Katrine Holde, Xiaoqin Liu, Trine Munk-Olsen, Kathrine Bang Madsen, Liselotte Vogdrup Petersen

**Affiliations:** National Centre for Register-based Research, Aarhus University, Aarhus, Denmark; National Centre for Register-based Research, Aarhus University, Aarhus, Denmark; and Department of Clinical Research, University of Southern Denmark, Odense, Denmark

**Keywords:** Mental disorders, low birthweight, neonatal outcomes, preterm birth

## Abstract

**Background:**

Previous studies have indicated associations between maternal mental disorders and adverse birth outcomes; however, these studies mainly focus on certain types of mental disorders, rather than the whole spectrum.

**Aims:**

We aimed to conduct a broad study examining all maternal mental disorder types and adverse neonatal outcomes which is needed to provide a more complete understanding of the associations.

**Method:**

We included 1 132 757 liveborn singletons born between 1997 and 2015 in Denmark. We compared children of mothers with a past (>2 years prior to conception; *n* = 48 646), recent (2 years prior to conception and during pregnancy; *n* = 15 899) or persistent (both past and recent; *n =* 10 905) diagnosis of any mental disorder, with children of mothers with no mental disorder diagnosis before the index delivery (*n* = 1 057 307). We also considered different types of mental disorders. We calculated odds ratios and 95% CIs of low birthweight, preterm birth, small for gestational age, low Apgar score, Caesarean delivery and neonatal death.

**Results:**

Odds ratios for children exposed to past, recent and persistent maternal mental disorders suggested an increased risk for almost all adverse neonatal outcomes. Estimates were highest for children in the ‘persistent’ group for all outcomes, with the exception of the association between persistent maternal mental disorders and neonatal death (odds ratio 0.96, 0.62–1.48).

**Conclusions:**

Our study provides evidence for increased risk of multiple adverse neonatal outcomes among children of mothers with mental disorders, highlighting the need for close monitoring and support for women with mental disorders.

The onset of mental disorders tends to occur early in life. Among women, most mental disorders develop before or during the child-bearing years,^1^ and pregnant women are among those that are diagnosed with mental disorders. Mental disorders are common causes of morbidity among pregnant women. In addition to the impact of mental disorders on the mother, several studies have highlighted the impact on outcomes in the offspring. Two reviews^1,2^ report associations between maternal mental disorders and preterm birth, low birthweight, small for gestational age (SGA) and Caesarean delivery. Adverse neonatal outcomes are, in turn, associated with detrimental long-term developmental outcomes in the offspring, including behavioural problems and lower school performance.^3^ Although these associations vary with, for example, the degree of low birthweight and prematurity, the World Health Organization has highlighted preterm birth as the leading cause of perinatal and neonatal morbidity and mortality.^4^ Previous studies on neonatal outcomes have focused on depression,^5^ anxiety,^6^ schizophrenia^7,8^ and eating disorders.^9,10^ There is limited research on other disorders, including personality disorders and developmental/behavioural disorders, which can occur just as frequently as or more frequently than some of the aforementioned disorders. A broad study examining all mental disorder types would allow for comparison and provide a more complete understanding of the associations between maternal mental disorders and adverse neonatal outcomes.

In this study, we used Danish nationwide registers to investigate adverse neonatal outcomes among offspring whose mothers had been diagnosed with a mental disorder. Compared with children of mothers with no mental disorder diagnosis, we considered children of mothers with (i) a past mental disorder diagnosis (more than 2 years prior to conception), (ii) a recent mental disorder diagnosis (2 years prior to conception and during pregnancy) and (iii) a persistent mental disorder diagnosis (both past and recent diagnoses). We examined the risk of adverse outcomes associated with any mental disorder, as well as different types of mental disorders.

## Method

### Study population

We carried out a population-based cohort study using Danish registers. We identified all liveborn singletons born between 1997 and 2015 (*n* = 1 159 624) from the Danish Medical Birth Register (MBR). All residents in Denmark are assigned a unique identification number in the Danish Civil Registration System, which allows linkage of data between different registers.

**Fig. 1:**
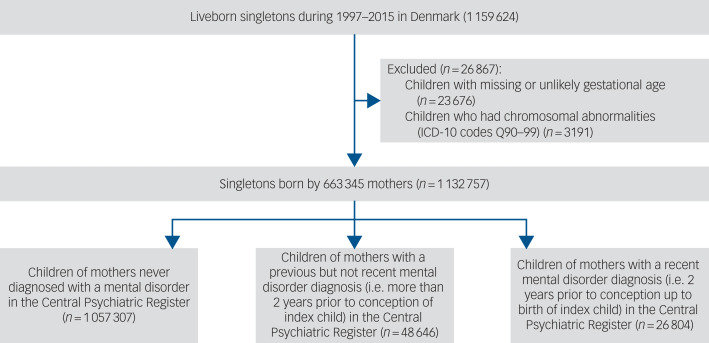
Flowchart showing identification of study population. ICD-10, International Classification of Diseases, 10th revision.

We excluded 23 676 children with missing or unlikely information on outcomes (gestational age <154 or >315 days, or missing; birthweight <300 or >6400 grams, or missing; missing Apgar score) and 3191 children with chromosomal abnormalities (ICD-10 [International Classification of Diseases, 10th revision] codes Q90–Q99) identified from the Danish National Patient Register. This resulted in a sample of 1 132 757 singletons born to 663 345 mothers (Fig. 1). The study was approved by the Danish Data Protection Agency. By Danish law, no informed consent is required for a register-based study using anonymised data. The research was carried out according to the Strengthening the Reporting of Observational Studies in Epidemiology (STROBE) guidelines (see Supplementary materials available at https://doi.org/10.1192/bjp.2024.164).

## Ethical approval

The study was approved by the Danish Data Protection Agency.

## Consent statement

By Danish law, no informed consent is required for a register-based study using anonymised data.

## Data sharing

No additional data are available. Access to individual-level Denmark data is governed by Danish authorities. These include the Danish Data Protection Agency, the Danish Health Data Authority, the Ethical Committee, and Statistics Denmark. Each scientific project must be approved before initiation, and approval is granted to a specific Danish research institution. Researchers at Danish research institutions may obtain the relevant approval and data. International researchers may gain data access if governed by a Danish research institution having needed approval and data access.

### Exposure: maternal mental disorder diagnosis

Maternal mental disorders were defined by ICD codes from in-patient, out-patient and emergency contacts in the Danish Psychiatric Central Research Register (PCRR) and Danish National Patient Register (NPR). The PCRR and NPR have recorded in-patient contacts since 1969 and 1977, respectively, as well as out-patient and emergency contacts since 1995. The main analyses examined any maternal mental disorder (ICD-10 codes F10–F99 and corresponding ICD-8 diagnoses [Supplementary Table 1]). Diagnoses were only included if they were made after disorder-dependent minimum ages of onset (Supplementary Table 1). We further classified mental disorders into exposure groups defined by nine ICD-10 subchapter categories (see Supplementary Table 1). We did not include organic mental disorders (ICD-10 codes F00–F09) as an exposure group as the onset of these disorders occur significantly later in life. Children whose mothers had never been diagnosed with a mental disorder before the index delivery were classified as unexposed (reference group). Children born to mothers who had been diagnosed with a mental disorder were divided into three groups: (i) past maternal mental disorder group: children born to a mother who had previously but not recently (more than 2 years prior to conception of the child) been diagnosed with a mental disorder, (ii) recent maternal mental disorder group: children born to a mother who had recently (between 2 years prior to conception and the birth of the child) been diagnosed with a mental disorder but did not have a history of mental disorder, (iii) persistent maternal mental disorder group: children born to a mother with both recent and past diagnoses (between 2 years prior to conception and the birth of the child, and more than 2 years prior to conception). Conception was ascertained from gestational age based on the first- or second-trimester ultrasound scan, or when ultrasound data were unavailable, the first day of the mother's last menstrual period.

An individual diagnosed with multiple mental disorders could be classified as exposed in more than one analysis.

### Outcomes: neonatal outcomes

We selected seven outcomes relating to labour, delivery and neonatal complications based on research highlighting these outcomes as associated with maternal mental disorders.^1,2^ Most outcomes were ascertained using the MBR: preterm birth (gestational age <37 weeks), low birthweight (birthweight <2500 g), SGA (i.e. birthweight <10th percentile for gender and gestational age), low 5-minute Apgar score (<7) and Caesarean delivery (surgery codes KMCA10-KMCA12). Neonatal deaths (death from any cause within 28 days of birth) were identified in the Danish Civil Registration System.

### Potential confounders

We adjusted for the following confounders in our main analyses: maternal age at delivery (<25, 25–34 or ≥35 years); parity (1, 2 or ≥3); maternal marital status at delivery (married/cohabiting or single/divorced/widowed); maternal highest education at delivery (elementary school/above elementary school); and calendar year of delivery (1997–2000, 2001–2005, 2006–2010 or 2011–2015). We additionally identified three pregnancy-related factors (Supplementary Fig. 1): number of non-psychiatric hospital visits during pregnancy (0–1, 2–3 or ≥4); smoking during pregnancy (yes/no); pregnancy complications (yes/no for gestational hypertension/eclampsia [ICD-10 O12–O15], diabetes mellitus during pregnancy [O24], pregnancy-related renal disease [O26.8] or antepartum haemorrhage [O46]). Data on these covariates were obtained from the aforementioned registers as well as Statistics Denmark's registers on socioeconomic status.

### Statistical analysis

Using R version 4.1.1 for Windows, we used logistic regression to calculate odds ratios with 95% CIs. In the main analyses, we adjusted for maternal age at delivery, parity, marital status at delivery, highest education at delivery and calendar year of delivery. As the two pregnancy-related factors could potentially be on the causal pathway, we adjusted for these in an additional analysis. To account for dependence between siblings, we used robust variance estimators for correction of standard errors. The main analyses were repeated for each mental disorder type. For marital status, smoking and education covariates, 0.5, 3.3 and 3.4% of values were missing, respectively; we applied multivariate logistic regression imputation by chained equations with 20 imputations to impute missing values.

#### Additional analysis

We also considered stillbirth, identified in the MBR. It should be noted that while the denominator for the neonatal outcomes included all live born children, the denominator for the outcome of stillbirth was the sum of all registered stillbirths and live births.

In addition to the broad categorisation of the outcomes, we considered several of them in more detail to see if results varied with the outcomes. We considered preterm birth at <32 weeks and 32–26 weeks; low birth weight of <1500 g and 1500–2500 g; SGA in <3 percentile and 3–10 percentile; and emergent, planned and unspecified Caesarean delivery.

We stratified the analyses to investigate whether the risk of adverse outcomes varied according to whether the mother had been prescribed and redeemed psychotropic medication during pregnancy, defined by Anatomic Therapeutic Chemical codes (N05 Psycholeptics and N06 Psychoanaleptics) in the Danish National Prescription Register.

We carried out three sensitivity analyses to test the robustness of our results. First, we assessed the impact of maternal low birthweight (maternal birthweight <2500 g, obtained from the MBR) as a confounder. As there was a relatively high proportion of missing data on maternal birthweight (partly because some mothers were born before the start of the MBR in 1973), we restricted these analyses to children whose mothers had available birthweight data (*n* = 602 880) and compared odds ratios with and without the addition of maternal low birthweight as a confounder. Second, we restricted the study population to firstborn children only (*n* = 494 354) to compare outcomes in children with siblings to children without siblings, as it has previously been demonstrated that parity influences associations between maternal mental disorders and neonatal outcomes.^11^ Third, for the same reason, we restricted the population to one child per mother, selected at random (*n* = 663 345).

## Results

The characteristics of the mothers of children in the study population are shown in Table 1. Among the 1 132 757 children, 48 646 (4.3%) were born to mothers with past mental disorder diagnoses, 15 899 (1.4%) to mothers with recent mental disorder diagnoses and 10 905 (1.0%) to mothers with persistent mental disorder diagnoses. Compared to mothers without a mental disorder diagnosis, mothers in all exposure groups were more likely to be have a higher number of non-psychiatric hospital visits during pregnancy; be single, divorced or widowed; not complete education beyond elementary school; and to have been prescribed a psychotropic medication during pregnancy. Additionally, mothers with past diagnoses were more likely to have at least their third child. Mothers with recent or persistent diagnoses were more likely be younger and primiparous.

**Table 1:** Characteristics of study population according to exposure status, *n* (%)

	Unexposed group (*n* = 1 057 307)	Past mental disorders only (*n* = 48 646)	Recent mental disorders only (*n* = 15 899)	Persistent (past/recent) mental disorders (*n* = 10 905)
Characteristics				
Maternal age at delivery				
<25 years	133 819 (13%)	7709 (16%)	5078 (32%)	2740 (25%)
25–34 years	734 883 (70%)	30 994 (64%)	8703 (55%)	6369 (58%)
≥35 years	188 605 (18%)	9943 (20%)	2118 (13%)	1796 (16%)
Parity				
1	459 710 (43%)	20 686 (43%)	8295 (52%)	5663 (52%)
2	399 815 (38%)	17 894 (37%)	4636 (29%)	3338 (31%)
≥3	197 782 (19%)	10 066 (21%)	2968 (19%)	1904 (17%)
Number of non-psychiatric hospital visits during pregnancy				
0–1	500 446 (47%)	16 121 (33%)	4962 (31%)	2733 (25%)
2–3	416 038 (39%)	20 558 (42%)	6516 (41%)	4577 (42%)
≥4	140 823 (13%)	11 967 (25%)	4421 (28%)	3595 (33%)
Pregnancy complications	87 205 (8.2%)	6140 (13%)	2184 (14%)	1786 (16%)
Maternal smoking during pregnancy				
No	860 588 (81%)	34 335 (71%)	9945 (63%)	6373 (58%)
Yes	162 107 (15%)	12 749 (26%)	5346 (34%)	4177 (38%)
Missing	34 612 (3.3%)	1562 (3.2%)	608 (3.8%)	355 (3.3%)
Maternal marital status at delivery				
Single, divorced or widowed	74 143 (7%)	7391 (15%)	3638 (23%)	2786 (26%)
Married or cohabiting	977 656 (92%)	41 086 (84%)	12 183 (77%)	8907 (74%)
Missing	5508 (1%)	169 (<1%)	78 (<1%)	22 (<1%)
Maternal highest education at delivery				
Elementary school	184 975 (17%)	15 632 (32%)	6888 (43%)	5332 (49%)
Above elementary school	834 896 (79%)	32 209 (66%)	8539 (54%)	5365 (49%)
Missing	37 436 (4%)	805 (2%)	472 (3%)	208 (2%)
Calendar year of delivery				
1997–2000	243 698 (23%)	3814 (8%)	2666 (17%)	888 (8%)
2001–2005	290 634 (27%)	9086 (19%)	3894 (24%)	1838 (17%)
2006–2010	279 414 (26%)	15 992 (33%)	4634 (29%)	3359 (31%)
2011–2015	243 561 (23%)	19 754 (41%)	4705 (30%)	4820 (44%)
Redeemed psychotropic prescriptions during pregnancy	17 621 (2%)	5489 (11%)	4352 (27%)	4325 (40%)
Sex of child				
Male	542 501 (51%)	24 888 (51%)	8223 (52%)	5557 (51%)
Female	514 806 (49%)	23 758 (49%)	7676 (48%)	5348 (49%)
Outcomes				
Preterm birth (<37 weeks)	50 579 (4.8%)	3037 (6.2%)	1135 (7.1%)	937 (8.6%)
Low birthweight (<2500 g)	35 115 (3.3%)	2236 (4.6%)	839 (5.3%)	697 (6.4%)
Small for gestational age	101 615 (9.6%)	5416 (11%)	1994 (13%)	1473 (14%)
Apgar score at 5 min	7263 (0.7%)	378 (0.8%)	150 (0.9%)	141 (1.3%)
Caesarean section	191 614 (18%)	10 614 (22%)	3291 (21%)	2505 (23%)
Neonatal death	1966 (0.2%)	91 (0.2%)	40 (0.3%)	21 (0.2%)

The numbers and proportions of each outcome are shown in Table 2. The most common mental disorders among mothers were neurotic disorders (2.6% of children had a mother with a past diagnosis; 1.0% recent; 0.3% persistent) and mood disorders (1.2% past; 0.6% recent; 0.2% persistent).

**Table 2:** Exposure status of the children in the study population by type of maternal mental disorder

Maternal mental disorder type	Exposure status – Maternal mental disorders			
	No mental disorders	Past only mental disorders	Recent only mental disorders	Persistent (past and recent) mental disorders
Any mental disorder	1 057 307 (93.3%)	48 646 (4.3%)	15 899 (1.4%)	10 905 (1.0%)
Mental and behavioural disorders due to psychoactive substance use Includes use of alcohol, cannabis, cocaine, nicotine, opioids, sedatives, hypnotics, anxiolytics, etc.	1 127 334 (99.5%)	3675 (0.3%)	1296 (0.1%)	452 (<0.1%)
Schizophrenia and related disorders Includes schizophrenia, schizotypal disorders, schizoaffective disorders and other psychotic disorders.	1 127 674 (99.6%)	3322 (0.3%)	931 (<0.1%)	830 (<0.1%)
Mood disorders Includes bipolar disorder, depressive disorders, etc.	1 110 590 (98.0%)	13 586 (1.2%)	6423 (0.6%)	2158 (0.2%)
Neurotic, stress-related and somatoform disorders Includes anxiety disorders, phobias, obsessive-compulsive disorders, etc.	1 088 108 (96.1%)	29 974 (2.6%)	11 131 (1.0%)	3544 (0.3%)
Eating disorders Includes anorexia nervosa, bulimia nervosa, etc.	1 121 189 (99.0%)	8713 (0.8%)	1611 (0.1%)	1244 (0.1%)
Personality disorders Includes paranoid personality disorder, etc.	1 114 493 (98.4%)	12 175 (1.1%)	4004 (0.4%)	2085 (0.2%)
Intellectual disabilities Includes mild to profound mental intellectual disability	1 132 144 (99.9%)	442 (<0.1%)	121 (<0.1%)	50 (<0.1%)
Developmental disorders Includes autism spectrum disorders	1 132 505 (100.0%)	191 (<0.1%)	35 (<0.1%)	26 (<0.1%)
Behavioural disorders Includes attention-deficit hyperactivity disorder, conduct disorders, childhood emotional disorders, etc.	1 126 894 (99.5%)	4768 (0.4%)	724 (<0.1%)	371 (<0.1%)

See Supplementary Appendix p. 4 for details of the International Classification of Diseases (ICD)-8 and ICD-10 codes used to define each mental disorder.

### Main analyses

**Fig. 2:**
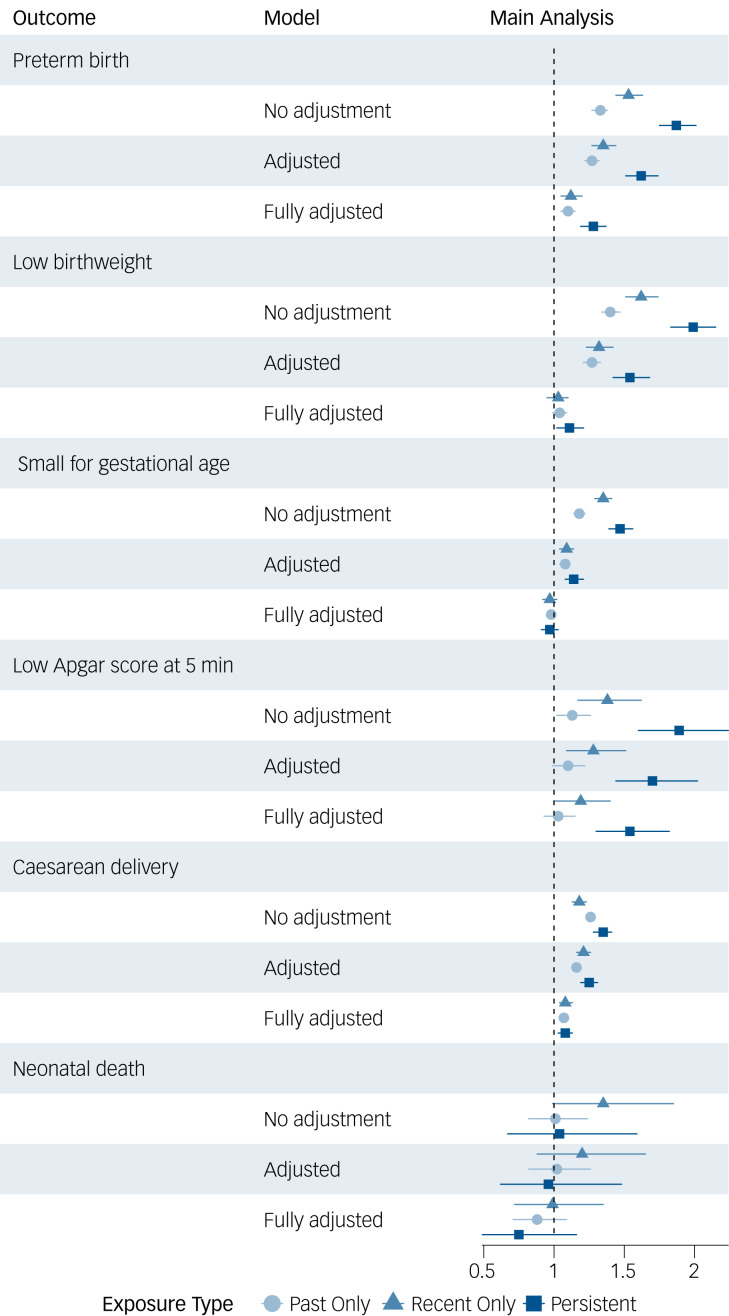
Adjusted odds ratios for the associations between maternal mental disorder diagnoses and birth outcomes. Crude: no adjustments. Adjusted: adjusted for maternal age at delivery; marital status; highest education; calendar year of delivery. Fully adjusted: adjusted for maternal age at delivery; marital status; highest education; calendar year of delivery; number of non-psychiatric hospital visits during pregnancy; smoking during pregnancy; pregnancy complications.

The risk of all adverse neonatal outcomes was elevated for children of mothers with either past, recent or persistent mental disorders, compared to the reference group, with the exception of neonatal death (Fig. 2 and Supplementary Table 2). For preterm birth, low birthweight, SGA, low Apgar score and Caesarean delivery, the risks followed the same pattern (to varying extents): highest among children of mothers with a persistent diagnosis, followed by children of mothers with a recent diagnosis and then by children of mothers with a past diagnosis. For example, the adjusted odds ratio for preterm birth was 1.62 (95% CI 1.51–1.74) for children of mothers with a persistent disorder, 1.35 (95% CI 1.27–1.44) for children of mothers with a recent disorder, and 1.27 (95% CI 1.22–1.32) for children of mothers with a past diagnosis. Adding the pregnancy-related factors resulted in attenuated odds ratios, with the odds ratios for SGA and neonatal death suggesting reduced risk (Fig. 2 and Supplementary Table 2).

For neonatal death, a different pattern was observed. The adjusted odds ratio was highest for children of mothers with a recent diagnosis (1.20, 95% CI 0.88–1.65), followed by children of mothers with a past diagnosis (1.02, 95% CI 1.51–1.74). It was slightly reduced for children of mothers with a persistent mental disorder (0.96, 95% CI 0⋅62–1.48). Statistical significance was not reached following any type of exposure.

### Analyses by mental disorder type

Results by mental disorder type can be found in the Supplementary Table 3. No consistent patterns emerged across mental disorders or outcomes. For past maternal diagnoses, adjusted models demonstrated that the strongest association was observed for development disorders and low Apgar score (2.23, 95% CI 0.55–8⋅97). For recent maternal diagnoses, the strongest association was observed for schizophrenia and low Apgar score (CI 1.90, 95% CI 0.90–4.01). For persistent maternal diagnoses, the strongest association was observed for substance use disorders and low Apgar score (3.22, 95% CI 1.43–7.23). Reduced odds were seen for several pairs, with statistical significance reached for Caesarean delivery for children whose mothers had a past eating disorder (0.91, 95% CI 0⋅85–0.98)

### Additional analyses

Supplementary Table 4 shows there were 4520 stillbirths in the period of interest. The risk of stillbirth was increased for recent mental disorders (odds ratio 1.25, 95% CI 1.01–1.55), but statistical significance was not reached for past or persistent mental disorders.

Considering the outcomes in more detail suggested some differences in risks (Supplementary Table 5). For example, the odds ratio for more extreme preterm birth (odds ratio 1.37, 95% CI 1.25–1.51) was higher than the risk for preterm birth at 32–36 weeks (1.28, 95% CI 1.21–1.35) among those with past mental disorders only. However the odds ratios for preterm birth at 32–36 weeks gestational age took higher values among those with recent or persistent disorders. Odds ratios were slightly higher for planned Caesarean delivery than emergent Caesarean delivery across exposure groups.

We observed some differences in associations when the sample was stratified by maternal psychotropic medication use during pregnancy (Supplementary Fig. 2 and Table 6). For example, among children in the recent exposure group whose mothers had taken psychotropic medication during pregnancy, there was an odds ratio of 1.60 (95% CI 0.90–2.86) for neonatal death; for those whose mothers had not taken psychotropic medication during pregnancy, the odds ratio was 0.98 (95% CI 0.65–1.46). For the persistent group, the pattern was in the opposite direction (0.62, 95% CI 0.26–1.48 for those whose mothers did take psychotropic medication during pregnancy versus 1.11, 95% CI 0.67–1.86 for those whose mothers did not take psychotropic medication during pregnancy).

The addition of maternal low birthweight as a potential confounder changed the results minimally (Supplementary Table 7 and Fig. 3).

Most adjusted odds ratios were all slightly reduced when restricted to firstborn children (Supplementary Fig. 3 and Table 8). For neonatal death following past diagnoses, the direction of association changed (from elevated risk in the main analysis to reduced risk in this analysis); for recent diagnoses, the odds ratio was further increased. When the population was limited to one child per mother, selected at random, several point estimates were higher than they were for the main analysis for children exposed to past or persistent mental disorders (Supplementary Table 9).

## Discussion

We found that the risk of preterm birth, low birthweight, SGA, low Apgar score, Caesarean delivery and neonatal death was increased among children in any of the exposure groups: any past, recent or persistent maternal mental disorder diagnoses. For these outcomes, we observed higher point estimates among children whose mothers had a persistent diagnosis than among children whose mothers had a past or recent diagnosis. However, neonatal death was increased only among children whose mothers had a past or recent diagnosis only, not persistent diagnoses, which appears contradictory.

When the sample was stratified by maternal psychotropic medication use during pregnancy, for most outcomes, we observed increased risk among mothers with recent or persistent diagnoses who had not been prescribed medication compared with mothers with recent or persistent diagnoses who had been prescribed medication. These results could reflect mothers with potentially active and untreated mental disorders or discontinuation of psychotropic medication. We also note there was between-group variation in sample size, and thus the prevalence of outcomes when the sample was stratified by medication use. Sensitivity analyses indicate some differences in odds ratios, though there is no identifiable pattern of change in results across exposure groups or outcomes.

### Comparison with previous literature

While there were no consistent patterns by mental disorder types, our results support those from studies indicating that some maternal mental disorders are associated with adverse neonatal outcomes.^1,2^ The grouping of diagnoses in this study into past, recent and persistent makes direct comparison with past research challenging; however, it is possible to identify some differences and similarities. Kelly et al^12^ reported that odds ratios for preterm birth and low birthweight were 1.6 (95% CI 1.4–1.9) and 2.0 (95% CI 1⋅7–2.3), respectively, among children born to mothers with psychiatric disorders, which are higher than odds ratios found in our study. Unlike our study, Kelly et al's definition of psychiatric disorders did not include substance use disorders, which were examined separately and revealed even higher odds ratios. Yin et al^13^ reported an odds ratio of 1.31 (95% CI 1.28–1.34) for maternal psychiatric diagnoses and preterm birth, which was higher than we observed for past diagnoses, but lower than we observed for recent or persistent diagnoses.

Our results indicate similar increases in risks for preterm birth and low birthweight compared with studies on substance use disorders,^12,14^ schizophrenia,^7,8,15^ mood disorders^5,16^ and eating disorders.^10^ Risks of preterm birth and low birthweight were found to be higher in children exposed to maternal personality disorders but to a slightly lower extent than previous studies.^17,18^ Consistent with Jablensky et al,^15^ we found an increased risk of low Apgar score in children exposed to maternal schizophrenia. For personality disorders, odds ratios for low Apgar score in this study were below Marshall et al's^17^ pooled effect estimate. For mood disorders^15,16^ and eating disorders,^9,10^ the risk of low Apgar score in our study fell within or close to ranges observed in previous studies. For recent diagnoses of any mental disorder, the risk of low Apgar score was similar to a recent Danish study by Heuckendorff et al^11^ (1.49, 95% CI 1.04–1.58), which examined mothers with mental disorders treated in primary care in the year before childbirth. Though the population in our study overlaps with Heuckendorff et al, we have provided novel findings regarding pre-conception maternal diagnoses, including persistent diagnoses.

Risks for Caesarean delivery were less elevated in our study compared with previous studies on schizophrenia^7,8,19^ and personality disorders.^18^ For intellectual disability, we found reduced risk of Caesarean delivery, whereas an increased risk has previously been reported.^20^ For eating disorders, the reduced risk we observed for Caesarean delivery was contrary to some previous findings,^10^ but similar to others.^9^

For neonatal death, our odds ratios fell around the estimates of previous studies for substance use^14^ and mood disorder.^16^ For schizophrenia and eating disorders, we found a reduced risk of neonatal death. A previous study has also indicated reduced risk in eating disorders,^9^ whereas previous studies consistently demonstrate increased risk in schizophrenia.^7,8,19^ It should be noted that neonatal death is the rarest outcome of those studied, and the observed odds ratios would result in a small increase in absolute terms (i.e. neonatal deaths per 100 000 live births: 186 in mothers with no diagnoses, 187 in mothers with past diagnoses and 228 in mothers with recent diagnoses).

Mental disorders are associated with various risk factors, including risky health behaviours.^21^ Several of these factors are also associated with adverse outcomes and may mediate associations between maternal mental disorders and neonatal outcomes. Prenatal exposure to tobacco smoke is an obvious covariate of interest. Women with mental disorders are more likely to continue smoking throughout pregnancy than women without mental disorders.^22^ The effect of smoking during pregnancy is clear, with evidence consistent that it increases the risk of a range of adverse birth outcomes.^23^ Alternatively, these associations may be modified by medication use.^24^ While we were able to adjust for maternal smoking during pregnancy and some other relevant factors (e.g. education attainment), we do not have data on all potentially relevant covariates in the Danish registers (e.g. binge drinking).

### Strengths and limitations

This study used Danish register data, which provide nationwide coverage and minimise selection and reporting biases. Diagnoses in the Danish registers have been validated for several mental disorders. These analyses provide a comprehensive overview of neonatal outcomes among children of mothers with various mental disorders, which addresses a key knowledge gap. Additionally, this study provides a clinically meaningful examination of the impact of past versus recent versus persistent diagnoses.

There are several limitations that should be considered. First, we relied on register-based diagnoses of mental disorders which reflect individuals who seek treatment in a hospital setting, and thus likely represent more severe cases. Diagnosis date is used as a proxy for mental disorder onset, though it may not reflect true onset. There is no information within Danish registers regarding remission; therefore, we do not know whether the absence of a recent diagnosis reflects a mother no longer requiring treatment or not seeking hospital treatment. Second, our study focused mainly on live births since we aimed to examine some postnatal outcomes. We did included stillbirth as an outcome, but covariate data is missing at a higher rate for stillbirths. Third, we were unable to include several potential confounders (e.g. planned versus unplanned pregnancy, or stigma from healthcare professionals) as they are not recorded within the Danish registers; these should be considered in future population-based studies. Fourth, we focused on the magnitude of estimates rather than statistical significance, but multiple comparisons may mean that some associations occurred due to chance. When adjusting for multiple comparisons, not all associations remained statistically significant. Fifth, we did not consider paternal mental disorders. A recent study in the Swedish population indicates that mental disorder diagnoses in fathers is associated with preterm birth.^13^ Follow-up studies could compare the impact of maternal versus paternal mental disorders on neonatal outcomes. Sixth, it was not within the scope of this study to consider associations between specific mental disorders and neonatal outcomes in more detail. Future studies could consider past and recent diagnoses of specific mental disorders in more detail, including stratification by specific medications relevant to each disorder. Finally, while our results support those reported in previous studies, the generalisability of our results outside Denmark is unclear.

In conclusion, the results have important research and clinical implications. Future studies should focus on examining the biological, psychological and social pathways that mediate associations between maternal mental disorders and adverse neonatal outcomes, such that targeted intervention can be implemented in perinatal and mental healthcare systems to improve outcomes among mothers and babies. The results indicate an elevated risk of several adverse outcomes not only in children of mothers with recent mental disorders, but also in children of mothers with past mental disorders. This highlights that mothers with any history of mental disorders, even those in remission, should receive additional support during the prenatal period. More broadly, this study underscores the need for improved prevention and early intervention of maternal mental disorders to mitigate adverse neonatal outcomes, especially in mothers with recent diagnoses.

Overall, this study provides evidence for increased risk of preterm birth, low birthweight, SGA, low Apgar score, Caesarean delivery and neonatal death among children of mothers with mental disorders diagnosed at most time points. These results underscore the need for additional follow-up of these women during the prenatal period. Further research is needed to identify modifiable factors that mediate associations between maternal mental disorders and adverse neonatal outcomes to highlight if, and when, intervention could potentially mitigate adverse outcomes.

## Supporting information

Momen et al. supplementary material Momen et al. supplementary material

## Data Availability

Access to individual-level Danish data is governed by Danish authorities. These include the Danish Data Protection Agency, the Danish Health Data Authority, the Ethical Committee and Statistics Denmark. Each scientific project must be approved before initiation, and approval is granted to a specific Danish research institution. Researchers at Danish research institutions may obtain the relevant approval and data. International researchers may gain data access if governed by a Danish research institution having needed approval and data access.
