# Supplementary material for: Maternal mental disorders and neonatal outcomes: Danish population-based cohort study
Source: Br J Psychiatry. 2025 Jan;226(1):31–8. doi: 10.1192/bjp.2024.164 (PMC11781859; doi:10.1192/bjp.2024.164)
Supplement: Momen et al. supplementary material [file S0007125024001648sup001.docx]

**Supplement**

**Maternal Mental Disorders and Neonatal Outcomes: a Danish Population-based Study**

Contents

[Supplementary Table 1. Mental disorders: International Classification of Disease (ICD) codes used for definition of cases among individuals born in Denmark between 1963 and 2010 2](#_Toc164179794)

[Supplementary Figure 1. Directed acyclic graph for 3](#_Toc164179795)

[Supplementary Table 2. Main analysis: Crude, adjusted, and fully adjusted odds ratios (ORs) with 95% confidence intervals (CIs) for the associations between maternal mental disorders and birth outcomes. 4](#_Toc164179796)

[Supplementary Table 3. Crude, adjusted, and fully adjusted odds ratios (ORs) with 95% confidence intervals (CIs) for the associations between different types of maternal mental disorders and birth outcomes. 5](#_Toc164179797)

[Supplementary Table 4. Main analysis: Crude, adjusted, and fully adjusted odds ratios (ORs) with 95% confidence intervals (CIs) for the associations between maternal mental disorders and stillbirth. 10](#_Toc164179798)

[Supplementary Figure 2. Crude, adjusted, and fully adjusted odds ratios (ORs) with 95% confidence intervals (CIs) for the associations between maternal mental disorders and birth outcomes in the main analysis (a) compared with the sensitivity analysis stratified by psychotropic medication use during pregnancy (b & c). 11](#_Toc164179799)

[Supplementary Table 5. Additional analysis: Crude, adjusted, and fully adjusted odds ratios (ORs) with 95% confidence intervals (CIs) for the associations between maternal mental disorders and birth outcomes, with more detailed definitions of preterm birth, low birthweight, small for gestational age and Caesarean delivery. 12](#_Toc164179800)

[Supplementary Table 6. Sensitivity analysis: Crude, adjusted, and fully adjusted odds ratios (ORs) with 95% confidence intervals (CIs) for the associations between maternal mental disorders and birth outcomes, stratified by recent psychotropic medication use (yes/no). 14](#_Toc164179801)

[Supplementary Table 7. Sensitivity analysis: Crude, adjusted, and fully adjusted odds ratios (ORs) with 95% confidence intervals (CIs) for the associations between maternal mental disorders and birth outcomes, including maternal low birthweight as a confounder. 16](#_Toc164179802)

[Supplementary Figure 3. Crude, adjusted, and fully adjusted odds ratios (ORs) with 95% confidence intervals (CIs) for the associations between maternal mental disorders and birth outcomes in the main analysis (a) compared with sensitivity analyses relating to firstborn children only (b) and maternal low birthweight (LBW) as a potential confounder (c). 17](#_Toc164179803)

[Supplementary Table 8. Sensitivity analysis: Crude, adjusted, and fully adjusted odds ratios (ORs) with 95% confidence intervals (CIs) for the associations between maternal mental disorders and birth outcomes, among firstborn children only. 19](#_Toc164179804)

[Supplementary Table 9. Sensitivity analysis: Crude, adjusted, and fully adjusted odds ratios (ORs) with 95% confidence intervals (CIs) for the associations between maternal mental disorders and birth outcomes, with the population restricted to one random birth per mother. 20](#_Toc164179805)

# Supplementary Table 1. Mental disorders: International Classification of Disease (ICD) codes used for definition of cases among individuals born in Denmark between 1963 and 2010

| **Disorder** | **Definition** | | |
| --- | --- | --- | --- |
|  | **ICD-10** | **ICD-8 equivalent** | **Minimum age**  **at diagnosis**  **(years)^a^** |
| **Substance use disorders** | F10-F19 | 291·x9, 294·39, 303·x9, 303·20, 303·28, 303·90, 304·x9 | 10 |
| **Schizophrenia spectrum disorders** | F20-F29 | 295·x9, 296·89, 297·x9, 298·29-298·99, 299·04, 299·05, 299·09, 301·83 | 10 |
| **Mood disorders** | F30-F39 | 296·x9 (excluding 296·89), 298·09, 298·19, 300·49, 301·19 | 10 |
| **Neurotic disorders** | F40-F48 | 300·x9 (excluding 300·49), 305·x9, 305·68, 307·99 | 5 |
| **Eating disorders** | F50 | 306·50, 306·58, 306·59 | 6 |
| **Personality disorders** | F60 | 301·x9 (excluding 301·19), 301·80, 301·81, 301·82, 301·84 | 10 |
| **Intellectual disability** | F70-F79 | 311·xx, 312·xx, 313·xx, 314·xx, 315·xx | 1 |
| **Developmental disorders** | F84 | 299·00, 299·01, 299·02, 299·03 | 1 |
| **Behavioral disorders** | F90-F98 | 306·x9, 308·0x | 1 |

^a^Minimum ages of onset, as specified in Pedersen CB, Mors O, Bertelsen A, Waltoft BL, Agerbo E, McGrath JJ, et al. A comprehensive nationwide study of the incidence rate and lifetime risk for treated mental disorders. JAMA Psychiatry. 2014;71(5):573-81.

# Supplementary Figure 1. Directed acyclic graph for


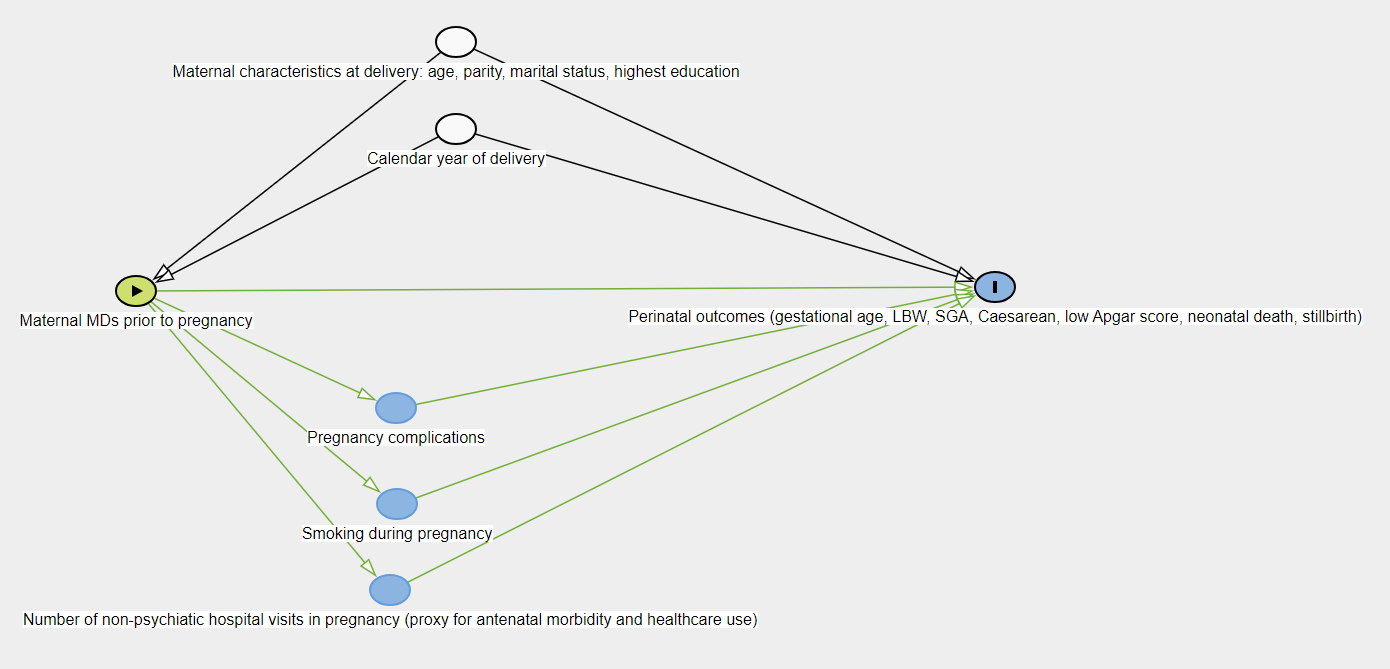


# Supplementary Table 2. Main analysis: Crude, adjusted, and fully adjusted odds ratios (ORs) with 95% confidence intervals (CIs) for the associations between maternal mental disorders and birth outcomes.

| **Outcome** | **No mental disorders**  **(N = 1,057,307)** | **Past only mental disorders**  **(N = 48,646)** | | | | **Recent only mental disorders**  **(N = 15,899)** | | | | **Persistent mental disorders**  **(N = 10,905)** | | | |
| --- | --- | --- | --- | --- | --- | --- | --- | --- | --- | --- | --- | --- | --- |
|  | **Cases (%)** | **Cases (%)** | **Crude** | **Adjusted** | **Fully adjusted** | **Cases (%)** | **Crude** | **Adjusted** | **Fully adjusted** | **Cases (%)** | **Crude** | **Adjusted** | **Fully adjusted** |
| **Preterm birth (<37 weeks)** | 50,579 (4‧8%) | 3,037 (6‧2%) | **1·33 (1·27-1·38)** | **1·27 (1·22-1·32)** | **1·10 (1·05-1·15)** | 1,135 (7‧1%) | **1·53 (1·44-1·63)** | **1·35 (1·27-1·44)** | **1·12 (1·05-1·2)** | 937 (8‧6%) | **1·87 (1·75-2·01)** | **1·62 (1·51-1·74)** | **1·28 (1·19-1·37)** |
| **Low birthweight (<2500 g)** | 35,115 (3‧3%) | 2,236 (4‧6%) | **1·40 (1·34-1·47)** | **1·27 (1·21-1·33)** | 1·04 (0·99-1·09) | 839 (5‧3%) | **1·62 (1·51-1·74)** | **1·32 (1·23-1·42)** | 1·03 (0·95-1·10) | 697 (6‧4%) | **1·99 (1·83-2·15)** | **1·54 (1·42-1·68)** | **1·11 (1·02-1·21)** |
| **Small for gestational age** | 101,615 (9‧6%) | 5,416 (11%) | **1·18 (1·14-1·22)** | **1·08 (1·04-1·11)** | 0·98 (0·95-1·01) | 1,994 (13%) | **1·35 (1·29-1·41)** | **1·09 (1·04-1·14)** | 0·97 (0·92-1·02) | 1,473 (14%) | **1·47 (1·39-1·56)** | **1·14 (1·08-1·21)** | 0·97 (0·91-1·03) |
| **Apgar score at 5 minutes (< 7)** | 7,263 (0‧7%) | 378 (0‧8%) | **1·13 (1·02-1·26)** | 1·10 (0·99-1·22) | 1·03 (0·93-1·15) | 150 (0‧9%) | **1·38 (1·17-1·62)** | **1·28 (1·09-1·51)** | **1·19 (1·01-1·4)** | 141 (1‧3%) | **1·89 (1·60-2·24)** | **1·7 (1·44-2·02)** | **1·54 (1·30-1·82)** |
| **Caesarean delivery** | 191,614 (18%) | 10,614 (22%) | **1·26 (1·23-1·29)** | **1·16 (1·13-1·19)** | **1·07 (1·04-1·1)** | 3,291 (21%) | **1·18 (1·13-1·23)** | **1·21 (1·16-1·26)** | **1·08 (1·04-1·13)** | 2,505 (23%) | **1·35 (1·28-1·41)** | **1·25 (1·19-1·31)** | **1·08 (1·03-1·13)** |
| **Neonatal death** | 1,966 (0‧2%) | 91 (0‧2%) | 1·01 (0·82-1·24) | 1·02 (0·82-1·26) | 0·88 (0·71-1·09) | 40 (0‧3%) | 1·35 (0·99-1·85) | 1·20 (0·88-1·65) | 0·99 (0·72-1·35) | 21 (0‧2%) | 1·04 (0·67-1·59) | 0·96 (0·62-1·48) | 0·75 (0·49-1·16) |

**Crude**: No adjustments.

**Adjusted**: Adjusted for maternal age at delivery; parity; marital status; highest education; calendar year of delivery.

**Fully adjusted**: Adjusted for maternal age at delivery; parity; marital status; highest education; calendar year of delivery; number of non-psychiatric hospital visits during pregnancy; smoking during pregnancy, pregnancy complications.

# Supplementary Table 3. Crude, adjusted, and fully adjusted odds ratios (ORs) with 95% confidence intervals (CIs) for the associations between different types of maternal mental disorders and birth outcomes.

| **Mental disorder** | **Outcome** | **No mental disorder**  **(N = 1,057,307)** | **Past only mental disorders**  **(N = 48,646)** | | | | **Recent only mental disorders**  **(N = 15,899)** | | | | **Persistent mental disorders**  **(N = 10,905)** | | | |
| --- | --- | --- | --- | --- | --- | --- | --- | --- | --- | --- | --- | --- | --- | --- |
|  |  | **Cases (%)** | **Cases (%)** | **Crude** | **Adjusted** | **Fully adjusted** | **Cases (%)** | **Crude** | **Adjusted** | **Fully adjusted** | **Cases (%)** | **Crude** | **Adjusted** | **Fully adjusted** |
| **Substance use disorder** | **Preterm birth (<37 weeks)** | 55,227 (4·9%) | 287 (7·8%) | **1·64 (1·45-1·87)** | **1·35 (1·15-1·59)** | 1·16 (0·98-1·37) | 116 (9·0%) | **1·91 (1·58-2·31)** | 1·30 (0·99-1·72) | 1·12 (0·84-1·49) | 58 (13%) | **2·86 (2·16-3·78)** | **1·72 (1·12-2·66)** | 1·30 (0·82-2·04) |
|  | **Low birthweight (<2500 g)** | 38,437 (3·4%) | 268 (7·3%) | **2·23 (1·95-2·55)** | **1·72 (1·44-2·05)** | **1·29 (1·07-1·55)** | 125 (9·6%) | **3·02 (2·51-3·64)** | **1·74 (1·31-2·29)** | 1·29 (0·97-1·72) | 57 (13%) | **4·09 (3·08-5·42)** | **2·67 (1·79-3·98)** | **1·69 (1·11-2·57)** |
|  | **Small for gestational age** | 109,515 (9·7%) | 605 (16%) | **1·83 (1·66-2·02)** | **1·54 (1·36-1·75)** | **1·21 (1·07-1·37)** | 275 (21%) | **2·50 (2·18-2·87)** | **1·54 (1·26-1·88)** | 1·16 (0·95-1·41) | 103 (23%) | **2·74 (2·19-3·43)** | **1·75 (1·25-2·47)** | 1·21 (0·86-1·70) |
|  | **Apgar score at 5 minutes (< 7)** | 7,864 (0·7%) | 39 (1·1%) | **1·53 (1·10-2·11)** | 0·91 (0·56-1·49) | 0·85 (0·52-1·40) | 20 (1·5%) | **2·23 (1·43-3·47)** | 1·25 (0·59-2·64) | 1·18 (0·56-2·49) | 9 (2·0%) | **2·89 (1·49-5·60)** | **3·22 (1·43-7·24)** | **2·83 (1·26-6·39)** |
|  | **Caesarean delivery** | 206,843 (18%) | 836 (23%) | **1·31 (1·20-1·43)** | **1·16 (1·03-1·30)** | 1·08 (0·96-1·22) | 227 (18%) | 0·94 (0·82-1·09) | 0·80 (0·64-1‧00) | **0·73 (0·59-0·92)** | 118 (26%) | **1·57 (1·27-1·95)** | 1·14 (0·81-1·60) | 0·96 (0·68-1·36) |
|  | **Neonatal death** | 2,103 (0·2%) | 10 (0·3%) | 1·46 (0·78-2·72) | 1·45 (0·69-3·03) | 1·24 (0·59-2·61) | 0-4 | NA | NA | NA | 0-4 | NA | NA | NA |
| **Schizophrenia** | **Preterm birth (<37 weeks)** | 55,287 (4·9%) | 245 (7·4%) | **1·54 (1·34-1·78)** | 1·13 (0·93-1·38) | 1·01 (0·83-1·23) | 78 (8·4%) | **1·77 (1·41-2·24)** | **1·52 (1·10-2·10)** | 1·32 (0·94-1·84) | 78 (9·4%) | **2·01 (1·57-2·58)** | 1·35 (0·90-2·03) | 1·16 (0·76-1·76) |
|  | **Low birthweight (<2500 g)** | 38,603 (3·4%) | 179 (5·4%) | **1·61 (1·37-1·88)** | 1·10 (0·88-1·38) | 0·92 (0·74-1·16) | 58 (6·2%) | **1·87 (1·43-2·45)** | 1·15 (0·75-1·76) | 0·96 (0·62-1·48) | 47 (5·7%) | **1·69 (1·22-2·35)** | 1·37 (0·85-2·20) | 1·10 (0·68-1·78) |
|  | **Small for gestational age** | 109,857 (9·7%) | 401 (12%) | **1·27 (1·14-1·42)** | 1·07 (0·93-1·24) | 0·97 (0·84-1·12) | 126 (14%) | **1·45 (1·20-1·75)** | 1·14 (0·88-1·49) | 1·06 (0·81-1·38) | 114 (14%) | **1·48 (1·20-1·81)** | 0·97 (0·70-1·35) | 0·84 (0·60-1·18) |
|  | **Apgar score at 5 minutes (< 7)** | 7,870 (0·7%) | 36 (1·1%) | **1·56 (1·12-2·16)** | 1·42 (0·92-2·18) | 1·35 (0·88-2·08) | 13 (1·4%) | **2·01 (1·17-3·48)** | 1·90 (0·90-4·01) | 1·78 (0·84-3·76) | 13 (1·6%) | **2·26 (1·31-3·91)** | 1·59 (0·66-3·83) | 1·49 (0·62-3·58) |
|  | **Caesarean delivery** | 206,821 (18%) | 772 (23%) | **1·35 (1·23-1·48)** | 1·13 (1‧00-1·29) | 1·07 (0·94-1·21) | 197 (21%) | **1·19 (1·02-1·40)** | 1·10 (0·87-1·39) | 1·01 (0·80-1·29) | 234 (28%) | **1·75 (1·49-2·05)** | **1·47 (1·14-1·88)** | **1·34 (1·04-1·72)** |
|  | **Neonatal death** | 2,107 (0·2%) | 6 (0·2%) | 0·97 (0·43-2·15) | 0·98 (0·37-2·62) | 0·88 (0·33-2·36) | 0-4 | NA | NA | NA | 0-4 | NA | NA | NA |
| **Mood disorder** | **Preterm birth (<37 weeks)** | 54,053 (4·9%) | 944 (6·9%) | **1·46 (1·36-1·57)** | **1·34 (1·23-1·46)** | **1·17 (1·08-1·28)** | 503 (7·8%) | **1·66 (1·51-1·82)** | **1·48 (1·32-1·66)** | **1·26 (1·12-1·41)** | 188 (8·7%) | **1·87 (1·60-2·17)** | **1·57 (1·29-1·91)** | **1·33 (1·09-1·62)** |
|  | **Low birthweight (<2500 g)** | 37,746 (3·4%) | 677 (5·0%) | **1·49 (1·37-1·62)** | **1·32 (1·20-1·46)** | 1·11 (1‧00-1·22) | 347 (5·4%) | **1·62 (1·46-1·81)** | **1·34 (1·17-1·53)** | 1·08 (0·93-1·24) | 117 (5·4%) | **1·63 (1·35-1·97)** | 1·25 (0·97-1·60) | 0·97 (0·75-1·26) |
|  | **Small for gestational age** | 100,059 (9·7%) | 1,469 (11%) | **1·12 (1·06-1·19)** | 1·02 (0·95-1·09) | 0·94 (0·88-1·01) | 717 (11%) | **1·17 (1·08-1·26)** | 0·97 (0·88-1·07) | **0·88 (0·80-0·97)** | 253 (12%) | **1·23 (1·08-1·41)** | 1·13 (0·96-1·34) | 0·99 (0·84-1·17) |
|  | **Apgar score at 5 minutes (< 7)** | 7,703 (0·7%) | 125 (0·9%) | **1·33 (1·11-1·59)** | 1·17 (0·94-1·45) | 1·10 (0·89-1·37) | 75 (1·2%) | **1·69 (1·35-2·13)** | **1·49 (1·11-1·98)** | **1·38 (1·04-1·85)** | 29 (1·3%) | **1·95 (1·35-2·81)** | **1·81 (1·15-2·85)** | **1·68 (1·07-2·65)** |
|  | **Caesarean delivery** | 202,819 (18%) | 3,204 (24%) | **1·38 (1·32-1·45)** | **1·18 (1·11-1·24)** | **1·09 (1·03-1·15)** | 1,469 (23%) | **1·33 (1·25-1·41)** | **1·26 (1·17-1·36)** | **1·14 (1·06-1·23)** | 532 (25%) | **1·46 (1·32-1·62)** | **1·23 (1·08-1·40)** | 1·11 (0·98-1·27) |
|  | **Neonatal death** | 2,077 (0·2%) | 19 (0·1%) | 0·75 (0·48-1·17) | 0·93 (0·57-1·52) | 0·82 (0·50-1·34) | 17 (0·3%) | 1·42 (0·88-2·28) | 1·64 (0·97-2·78) | 1·39 (0·82-2·36) | 5 (0·2%) | 1·24 (0·52-2·98) | 1·55 (0·58-4·14) | 1·33 (0·50-3·58) |
| **Neurotic disorder** | **Preterm birth (<37 weeks)** | 52,620 (4·8%) | 1,994 (6·7%) | **1·40 (1·34-1·47)** | **1·31 (1·24-1·38)** | **1·12 (1·06-1·18)** | 777 (7·0%) | **1·48 (1·37-1·59)** | **1·24 (1·14-1·35)** | 1·04 (0·95-1·13) | 297 (8·4%) | **1·80 (1·59-2·03)** | **1·49 (1·30-1·72)** | 1·14 (0·99-1·32) |
|  | **Low birthweight (<2500 g)** | 36,635 (3·4%) | 1,445 (4·8%) | **1·45 (1·37-1·54)** | **1·28 (1·20-1·36)** | 1·03 (0·96-1·10) | 582 (5·2%) | **1·58 (1·46-1·72)** | **1·24 (1·12-1·36)** | 0·98 (0·88-1·08) | 225 (6·3%) | **1·95 (1·70-2·23)** | **1·47 (1·26-1·73)** | 1·04 (0·89-1·22) |
|  | **Small for gestational age** | 105,333 (9·7%) | 3,305 (11%) | **1·16 (1·11-1·20)** | 1·03 (0·99-1·07) | 0·93 (0·89-0·97) | 1,401 (13%) | **1·34 (1·27-1·42)** | **1·10 (1·03-1·17)** | 0·99 (0·93-1·06) | 459 (13%) | **1·39 (1·26-1·54)** | 1·12 (1‧00-1·25) | 0·96 (0·85-1·07) |
|  | **Apgar score at 5 minutes (< 7)** | 7,521 (0·7%) | 260 (0·9%) | **1·26 (1·11-1·43)** | 1·15 (1‧00-1·33) | 1·08 (0·93-1·24) | 114 (1·0%) | **1·49 (1·23-1·79)** | 1·22 (0·98-1·53) | 1·13 (0·91-1·42) | 37 (1·0%) | **1·52 (1·10-2·10)** | 1·22 (0·83-1·80) | 1·08 (0·74-1·60) |
|  | **Caesarean delivery** | 197,832 (18%) | 6,968 (23%) | **1·36 (1·32-1·41)** | **1·24 (1·20-1·29)** | **1·13 (1·09-1·17)** | 2,361 (21%) | **1·21 (1·16-1·27)** | **1·26 (1·20-1·33)** | **1·13 (1·07-1·19)** | 863 (24%) | **1·45 (1·34-1·57)** | **1·32 (1·21-1·45)** | **1·12 (1·02-1·23)** |
|  | **Neonatal death** | 2,022 (0·2%) | 67 (0·2%) | 1·20 (0·94-1·53) | 1·20 (0·92-1·57) | 1·02 (0·78-1·33) | 25 (0·2%) | 1·21 (0·81-1·79) | 1·02 (0·65-1·61) | 0·84 (0·54-1·33) | 0-4 | NA | NA | NA |
| **Eating disorder** | **Preterm birth (<37 weeks)** | 54,913 (4·9%) | 530 (6·1%) | **1·26 (1·14-1·38)** | **1·19 (1·08-1·32)** | **1·14 (1·03-1·27)** | 133 (8·3%) | **1·75 (1·46-2·10)** | **1·51 (1·21-1·89)** | **1·34 (1·07-1·68)** | 112 (9·0%) | **1·92 (1·58-2·34)** | **1·68 (1·35-2·09)** | **1·49 (1·19-1·86)** |
|  | **Low birthweight (<2500 g)** | 38,323 (3·4%) | 370 (4·2%) | **1·25 (1·12-1·40)** | **1·17 (1·03-1·32)** | 1·11 (0·98-1·26) | 102 (6·3%) | **1·91 (1·55-2·35)** | **1·59 (1·24-2·05)** | **1·37 (1·06-1·77)** | 92 (7·4%) | **2·26 (1·80-2·83)** | **1·80 (1·39-2·32)** | **1·56 (1·20-2·03)** |
|  | **Small for gestational age** | 109,078 (9·7%) | 982 (11%) | **1·18 (1·10-1·27)** | **1·13 (1·05-1·22)** | **1·12 (1·04-1·21)** | 238 (15%) | **1·61 (1·40-1·85)** | **1·24 (1·05-1·48)** | 1·18 (0·99-1·41) | 200 (16%) | **1·78 (1·52-2·08)** | **1·38 (1·16-1·65)** | **1·33 (1·11-1·59)** |
|  | **Apgar score at 5 minutes (< 7)** | 7,848 (0·7%) | 52 (0·6%) | 0·85 (0·65-1·12) | 0·78 (0·58-1·05) | 0·77 (0·57-1·04) | 11 (0·7%) | 0·98 (0·51-1·86) | 0·79 (0·38-1·67) | 0·75 (0·36-1·58) | 21 (1·7%) | **2·44 (1·58-3·75)** | **1·73 (1·02-2·94)** | 1·64 (0·97-2·78) |
|  | **Caesarean delivery** | 205,811 (18%) | 1,656 (19%) | 1·04 (0·98-1·11) | 0·91 (0·85-0·98) | 0·88 (0·81-0·94) | 314 (19%) | 1·08 (0·95-1·22) | 1·04 (0·89-1·22) | 0·96 (0·82-1·12) | 243 (20%) | 1·08 (0·93-1·25) | 0·90 (0·76-1·07) | **0·82 (0·70-0·98)** |
|  | **Neonatal death** | 2,107 (0·2%) | 7 (<0·1%) | **0·43 (0·20-0·90)** | 0·45 (0·20-1·01) | **0·43 (0·19-0·95)** | 0-4 | NA | NA | NA | 0-4 | NA | NA | NA |
| **Personality disorder** | **Preterm birth (<37 weeks)** | 54,342 (4·9%) | 829 (6·8%) | **1·43 (1·32-1·54)** | **1·24 (1·12-1·37)** | 1·04 (0·94-1·15) | 328 (8·2%) | **1·74 (1·55-1·95)** | **1·39 (1·18-1·65)** | 1·12 (0·95-1·33) | 189 (9·1%) | **1·94 (1·67-2·26)** | **1·50 (1·19-1·89)** | 1·11 (0·87-1·41) |
|  | **Low birthweight (<2500 g)** | 37,875 (3·4%) | 614 (5·0%) | **1·51 (1·38-1·65)** | **1·23 (1·10-1·38)** | 0·97 (0·86-1·09) | 245 (6·1%) | **1·85 (1·63-2·11)** | **1·41 (1·17-1·69)** | 1·05 (0·87-1·27) | 153 (7·3%) | **2·25 (1·91-2·65)** | **1·41 (1·08-1·83)** | 0·94 (0·71-1·24) |
|  | **Small for gestational age** | 108,288 (9·7%) | 1,417 (12%) | **1·22 (1·15-1·30)** | **1·10 (1·02-1·19)** | 0·98 (0·91-1·06) | 508 (13%) | **1·35 (1·23-1·48)** | 0·95 (0·82-1·09) | 0·82 (0·71-0·94) | 285 (14%) | **1·47 (1·29-1·67)** | 1‧00 (0·82-1·22) | 0·83 (0·68-1·01) |
|  | **Apgar score at 5 minutes (< 7)** | 7,747 (0·7%) | 117 (1·0%) | **1·39 (1·15-1·67)** | 1·27 (1‧00-1·62) | 1·18 (0·93-1·51) | 39 (1·0%) | **1·41 (1·02-1·93)** | 1·49 (0·98-2·25) | 1·36 (0·90-2·05) | 29 (1·4%) | **2·02 (1·40-2·91)** | **1·83 (1·08-3·10)** | 1·61 (0·95-2·73) |
|  | **Caesarean delivery** | 203,968 (18%) | 2,798 (23%) | **1·33 (1·27-1·40)** | **1·18 (1·10-1·26)** | 1·07 (1‧00-1·14) | 758 (19%) | 1·04 (0·96-1·13) | 1·08 (0·97-1·22) | 0·95 (0·85-1·07) | 500 (24%) | **1·41 (1·27-1·56)** | **1·30 (1·11-1·52)** | 1·09 (0·93-1·28) |
|  | **Neonatal death** | 2,084 (0·2%) | 22 (0·2%) | 0·97 (0·63-1·47) | 0·89 (0·52-1·54) | 0·75 (0·44-1·30) | 10 (0·2%) | 1·34 (0·72-2·49) | 1·74 (0·86-3·49) | 1·41 (0·70-2·83) | 0-4 | NA | NA | NA |
| **Intellectual disability** | **Preterm birth (<37 weeks)** | 55,630 (4·9%) | 43 (9·7%) | **2·09 (1·50-2·91)** | 1·26 (0·76-2·09) | 1·11 (0·65-1·88) | 11 (9·1%) | **1·94 (1·04-3·61)** | 0·28 (0·04-2·01) | 0·24 (0·03-1·78) | 0-4 | NA | NA | NA |
|  | **Low birthweight (<2500 g)** | 38,848 (3·4%) | 25 (5·7%) | **1·69 (1·11-2·56)** | 0·84 (0·44-1·57) | 0·68 (0·36-1·27) | 11 (9·1%) | **2·81 (1·51-5·25)** | 1·42 (0·52-3·94) | 1·30 (0·44-3·77) | 0-4 | NA | NA | NA |
|  | **Small for gestational age** | 110,394 (9·8%) | 66 (15%) | **1·62 (1·24-2·13)** | 0·82 (0·54-1·26) | 0·72 (0·47-1·08) | 24 (20%) | **2·29 (1·45-3·61)** | 1·15 (0·57-2·33) | 1·14 (0·57-2·30) | 14 (28%) | **3·60 (1·91-6·77)** | 1·91 (0·71-5·13) | 1·69 (0·61-4·68) |
|  | **Apgar score at 5 minutes (< 7)** | 7,919 (0·7%) | 10 (2·3%) | **3·29 (1·66-6·52)** | 1·54 (0·49-4·84) | 1·46 (0·47-4·58) | 0-4 | NA | NA | NA | 0-4 | NA | NA | NA |
|  | **Caesarean delivery** | 207,905 (18%) | 85 (19%) | 1·06 (0·80-1·40) | 0·92 (0·62-1·36) | 0·85 (0·57-1·28) | 23 (19%) | 1·04 (0·66-1·65) | 0·50 (0·20-1·24) | 0·46 (0·18-1·18) | 11 (22%) | 1·25 (0·63-2·48) | 2·03 (0·76-5·41) | 1·88 (0·65-5·42) |
|  | **Neonatal death** | 2,117 (0·2%) | 0-4 | NA | NA | NA | 0 (0%) | 0 (0-0) | 0 (0-0) | 0 (0-0) | 0 (0%) | 0 (0-0) | 0 (0-0) | 0 (0-0) |
| **Developmental disorder** | **Preterm birth (<37 weeks)** | 55,672 (4·9%) | 14 (7·3%) | 1·53 (0·88-2·65) | 0·92 (0·37-2·27) | 0·85 (0·35-2·08) | 0-4 | NA | NA | NA | 0-4 | 0·77 (0·1-5·76) | 0 (0-0·01) | 0 (0-0) |
|  | **Low birthweight (<2500 g)** | 38,877 (3·4%) | 7 (3·7%) | 1·07 (0·51-2·24) | 0·56 (0·18-1·72) | 0·52 (0·18-1·56) | 0-4 | NA | NA | NA | 0-4 | 2·34 (0·55-10·06) | 0 (0-0) | 0 (0-0) |
|  | **Small for gestational age** | 110,466 (9·8%) | 22 (12%) | 1·2 (0·78-1·86) | 1·01 (0·59-1·73) | 1·02 (0·6-1·72) | 0-4 | NA | NA | NA | 7  (27%) | **3·41 (1·38-8·41)** | 2·64 (0·49-14·27) | 2·78 (0·45-17·29) |
|  | **Apgar score at 5 minutes (< 7)** | 7,925 (0·7%) | 5 (2·6%) | **3·81 (1·36-10·73)** | 2·23 (0·55-8·95) | 2·16 (0·53-8·75) | 0-4 | NA | NA | NA | 0-4 | NA | NA | NA |
|  | **Caesarean delivery** | 207,966 (18%) | 51 (27%) | **1·62 (1·09-2·40)** | 1·62 (0·96-2·75) | 1·58 (0·92-2·70) | 0-4 | NA | NA | NA | 0-4 | NA | NA | NA |
|  | **Neonatal death** | 2,118 (0·2%) | 0 (0%) | 0 (0-0) | 0 (0-0) | 0 (0-0) | 0 (0%) | 0 (0-0) | 0 (0-0) | 0 (0-0) | 0 (0%) | 0 (0-0) | 0 (0-0) | 0 (0-0) |
| **Behavioral disorder** | **Preterm birth (<37 weeks)** | 55,212 (4·9%) | 386 (8·1%) | **1·71 (1·53-1·92)** | **1·41 (1·24-1·60)** | **1·20 (1·06-1·37)** | 53 (7·3%) | **1·53 (1·15-2·04)** | 1·15 (0·76-1·73) | 0·90 (0·60-1·37) | 37 (10·0%) | **2·15 (1·52-3·04)** | **2·09 (1·41-3·11)** | **1·72 (1·14-2·59)** |
|  | **Low birthweight (<2500 g)** | 38,513 (3·4%) | 297 (6·2%) | **1·88 (1·64-2·14)** | **1·40 (1·21-1·63)** | 1·11 (0·95-1·29) | 49 (6·8%) | **2·05 (1·54-2·74)** | 1·12 (0·73-1·72) | 0·80 (0·51-1·24) | 28 (7·5%) | **2·31 (1·53-3·48)** | **1·77 (1·12-2·79)** | 1·30 (0·81-2·10) |
|  | **Small for gestational age** | 109,656 (9·7%) | 6658 (14%) | **1·49 (1·36-1·62)** | 1·11 (1‧00-1·22) | 0·96 (0·87-1·06) | 120 (17%) | **1·84 (1·52-2·24)** | 0·97 (0·73-1·30) | 0·81 (0·60-1·08) | 64 (1.3%) | **1·93 (1·47-2·54)** | 1·33 (0·96-1·84) | 1·08 (0·78-1·50) |
|  | **Apgar score at 5 minutes (< 7)** | 7,875 (0·7%) | 46 (1·0%) | **1·38 (1·03-1·86)** | **1·45 (1·07-1·97)** | 1·35 (1‧00-1·84) | 6 (0·8%) | 1·19 (0·53-2·65) | 0·93 (0·30-2·90) | 0·84 (0·27-2·63) | 5 (1·3%) | 1·94 (0·80-4·69) | 1·89 (0·70-5·07) | 1·72 (0·64-4·62) |
|  | **Caesarean delivery** | 206,825 (18%) | 974 (20%) | **1·14 (1·05-1·24)** | **1·16 (1·06-1·28)** | 1·07 (0·97-1·17) | 160 (22%) | **1·26 (1·06-1·51)** | 1·20 (0·92-1·55) | 1·06 (0·81-1·38) | 65 (18%) | 0·94 (0·71-1·25) | 0·86 (0·60-1·22) | 0·76 (0·53-1·09) |
|  | **Neonatal death** | 2,105 (0·2%) | 9 (0·2%) | 1·01 (0·53-1·94) | 0·95 (0·47-1·90) | 0·81 (0·40-1·63) | 0-4 | NA | NA | NA | 0-4 | NA | NA | NA |

**Crude:** No adjustments.

**Adjusted:** Adjusted for maternal age at delivery; parity; marital status; highest education; calendar year of delivery; prior comorbid mental disorders.

**Fully adjusted:** Adjusted for maternal age at delivery; parity; marital status; highest education; calendar year of delivery; prior comorbid mental disorders; number of non-psychiatric hospital visits during pregnancy; smoking during pregnancy, pregnancy complications.

# Supplementary Table 4. Main analysis: Crude, adjusted, and fully adjusted odds ratios (ORs) with 95% confidence intervals (CIs) for the associations between maternal mental disorders and stillbirth.

There were 4,520 stillbirths in the population (not possible to include as an outcome in the other analyses due to missing information on these births).

| **Outcome** | **No mental disorders**  **(N = 1,061,449)** | **Past only mental disorders**  **(N = 48,871)** | | | | **Recent only mental disorders**  **(N = 15,990)** | | | | **Persistent mental disorders**  **(N = 10,967)** | | | |
| --- | --- | --- | --- | --- | --- | --- | --- | --- | --- | --- | --- | --- | --- |
|  | **Cases (%)** | **Cases (%)** | **Crude** | **Adjusted** | **Fully adjusted** | **Cases (%)** | **Crude** | **Adjusted** | **Fully adjusted** | **Cases (%)** | **Crude** | **Adjusted** | **Fully adjusted** |
| **Stillbirth** | 4,142 (0‧4%) | 225 (0‧5%) | **1·18 (1·03-1·36)** | 1·10 (0·95-1·27) | 1·02 (0·89-1·18) | 91 (0‧6%) | **1·46 (1·18-1·80)** | **1·25 (1·01-1·55)** | 1·14 (0·92-1·41) | 62 (0‧6%) | **1·45 (1·12-1·87)** | 1·23 (0·94-1·59) | 1·08 (0·83-1·40) |

**Crude:** No adjustments.

**Adjusted:** Adjusted for maternal age at delivery; marital status; highest education; calendar year of delivery.

**Fully adjusted:** Adjusted for maternal age at delivery; marital status; highest education; calendar year of delivery; number of non-psychiatric hospital visits during pregnancy; smoking during pregnancy; pregnancy complications.

# Supplementary Figure 2. Crude, adjusted, and fully adjusted odds ratios (ORs) with 95% confidence intervals (CIs) for the associations between maternal mental disorders and birth outcomes in the main analysis (a) compared with the sensitivity analysis stratified by psychotropic medication use during pregnancy (b & c).


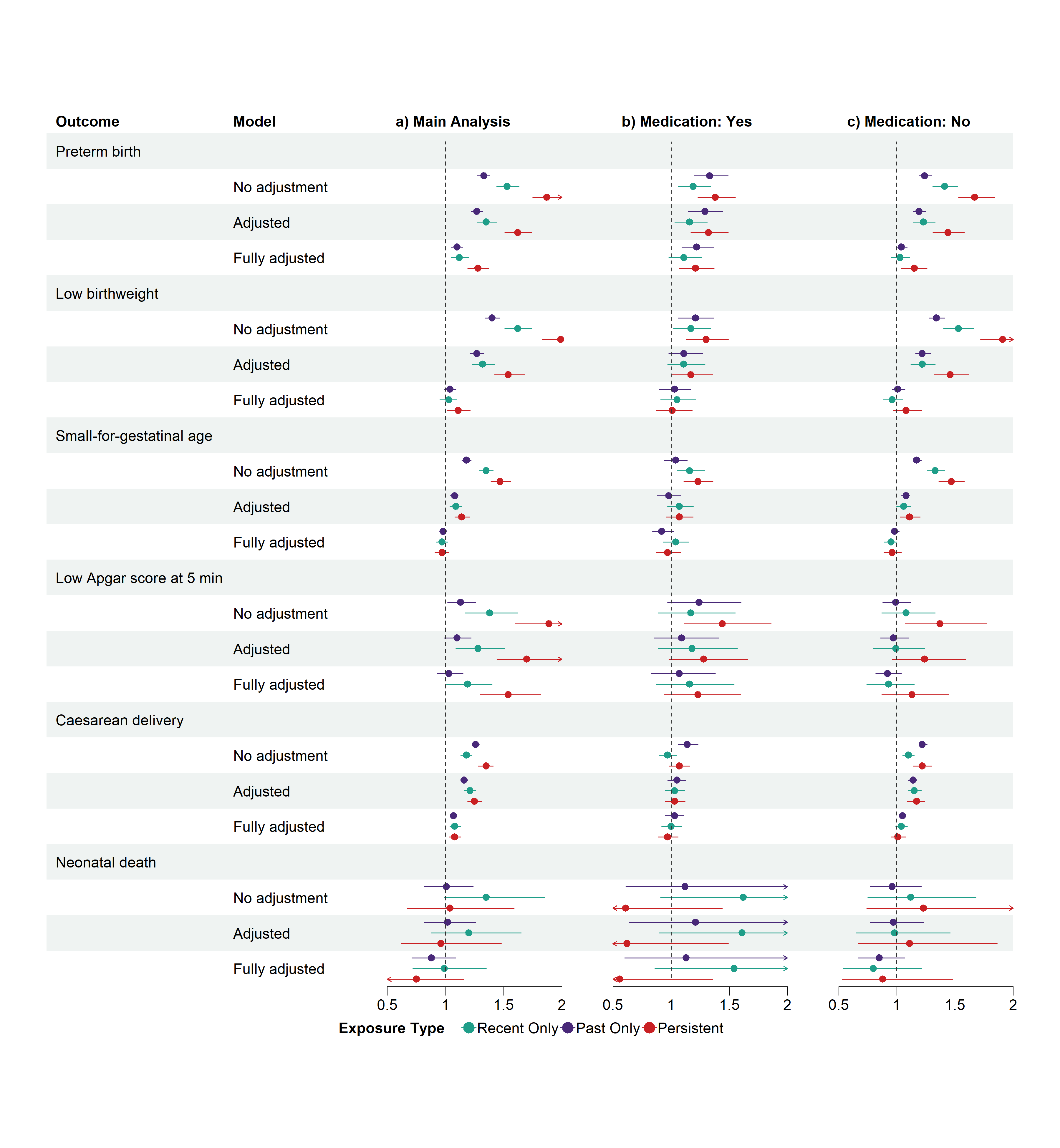


# Supplementary Table 5. Additional analysis: Crude, adjusted, and fully adjusted odds ratios (ORs) with 95% confidence intervals (CIs) for the associations between maternal mental disorders and birth outcomes, with more detailed definitions of preterm birth, low birthweight, small for gestational age and Caesarean delivery.

| **Outcome** |  | **No mental disorders**  **(N = 1,057,307)** | **Past only mental disorders**  **(N = 48,646)** | | | | **Recent only mental disorders**  **(N = 15,899)** | | | | **Persistent mental disorders**  **(N = 10,905)** | | | |
| --- | --- | --- | --- | --- | --- | --- | --- | --- | --- | --- | --- | --- | --- | --- |
|  |  | **Cases (%)** | **Cases (%)** | **Crude** | **Adjusted** | **Fully adjusted** | **Cases (%)** | **Crude** | **Adjusted** | **Fully adjusted** | **Cases (%)** | **Crude** | **Adjusted** | **Fully adjusted** |
| **Preterm birth** | <32 weeks | 7,104 (0‧7%) | 473 (1‧0%) | **1·46 (1·33-1·61)** | **1·37 (1·24-1·51)** | 1·10 (1‧00-1·21) | 150 (1‧0%) | **1·43 (1·21-1·68)** | **1·22 (1·03-1·44)** | 0·91 (0·77-1·08) | 116 (1‧1%) | **1·63 (1·35-1·96)** | **1·35 (1·12-1·63)** | 0·94 (0·78-1·14) |
|  | 32-36 weeks | 26,430 (2‧5%) | 1,589 (3‧3%) | **1·32 (1·25-1·39)** | **1·28 (1·21-1·35)** | **1·10 (1·05-1·17)** | 597 (3‧8%) | **1·53 (1·40-1·66)** | **1·35 (1·24-1·47)** | **1·11 (1·02-1·21)** | 521 (4‧8%) | **1·97 (1·79-2·15)** | **1·71 (1·56-1·88)** | **1·34 (1·22-1·47)** |
| **Low birthweight** | <1500g | 5,695 (0‧6%) | 393 (0‧8%) | **1·52 (1·37-1·69)** | **1·41 (1·27-1·56)** | 1·10 (0·99-1·22) | 120 (0‧8%) | **1·43 (1·19-1·71)** | 1·19 (0·99-1·43) | 0·85 (0·71-1·03) | 97 (0‧9%) | **1·71 (1·39-2·09)** | **1·36 (1·11-1·67)** | 0·9 (0·73-1·11) |
|  | 1500-2500g | 29,420 (2‧8%) | 1,843 (3‧8%) | **1·38 (1·31-1·45)** | **1·24 (1·18-1·31)** | 1·03 (0·98-1·08) | 719 (4‧6%) | **1·66 (1·54-1·79)** | **1·34 (1·24-1·45)** | 1·06 (0·98-1·15) | 600 (5‧6%) | **2·04 (1·87-2·23)** | **1·58 (1·45-1·72)** | **1·15 (1·05-1·26)** |
| **Small for gestational age** | <3% | 30,372 (3‧1%) | 1,633 (3‧6%) | **1·19 (1·13-1·25)** | **1·09 (1·03-1·15)** | 0·96 (0·91-1·01) | 639 (4‧4%) | **1·45 (1·33-1·57)** | **1·12 (1·03-1·22)** | 0·95 (0·88-1·04) | 494 (5‧0%) | **1·65 (1·50-1·81)** | **1·23 (1·12-1·36)** | 0·99 (0·90-1·09) |
|  | 3-10% | 71,243 (6‧9%) | 3,783 (8‧0%) | **1·17 (1·13-1·22)** | **1·07 (1·03-1·11)** | 0·99 (0·96-1·03) | 1,355 (8‧9%) | **1·31 (1·24-1·38)** | **1·07 (1·01-1·14)** | 0·98 (0·92-1·03) | 979 (9‧4%) | **1·39 (1·30-1·49)** | **1·10 (1·02-1·18)** | 0·96 (0·90-1·03) |
| **Caesarean delivery** | Emergent | 107,288 (11%) | 5,874 (13%) | **1·25 (1·21-1·29)** | **1·14 (1·11-1·18)** | **1·06 (1·03-1·10)** | 1,861 (13%) | **1·19 (1·13-1·25)** | **1·15 (1·09-1·21)** | 1·04 (0·99-1·10) | 1,425 (15%) | **1·37 (1·29-1·45)** | **1·19 (1·12-1·27)** | 1·04 (0·98-1·11) |
|  | Planned | 74,600 (7‧9%) | 4,448 (10%) | **1‧36 (1‧31-1‧41)** | **1‧20 (1‧15-1‧24)** | **1‧09 (1‧05-1‧14)** | 1,289 (9‧3%) | **1‧19 (1‧12-1‧26)** | **1‧33 (1‧25-1‧41)** | **1‧17 (1‧11-1‧25)** | 1,003 (11%) | **1‧39 (1‧29-1‧49)** | **1‧36 (1‧27-1‧46)** | **1‧17 (1‧09-1‧26)** |
|  | Unspecified | 9,726 (1‧1%) | 292 (0‧8%) | 0·68 (0·61-0·77) | 1·11 (0·98-1·25) | 0·96 (0·85-1·08) | 141 (1‧1%) | 1‧00 (0·84-1·18) | 1·17 (0·99-1·39) | 0·95 (0·80-1·12) | 77 (0‧9%) | 0·82 (0·65-1·02) | 1·30 (1·04-1·64) | 1·02 (0·81-1·28) |

**Crude:** No adjustments.

**Adjusted:** Adjusted for maternal age at delivery; marital status; highest education; calendar year of delivery.

**Fully adjusted:** Adjusted for maternal age at delivery; marital status; highest education; calendar year of delivery; number of non-psychiatric hospital visits during pregnancy; smoking during pregnancy; pregnancy complications.

# Supplementary Table 6. Sensitivity analysis: Crude, adjusted, and fully adjusted odds ratios (ORs) with 95% confidence intervals (CIs) for the associations between maternal mental disorders and birth outcomes, stratified by recent psychotropic medication use (yes/no).

| **Group** | **Outcome** | **No mental disorders**  **(N = 1,057,307)** | **Past only mental disorders**  **(N = 48,646)** | | | | | **Recent only mental disorders**  **(N = 15,899)** | | | | | **Persistent mental disorders**  **(N = 10,905)** | | | | |
| --- | --- | --- | --- | --- | --- | --- | --- | --- | --- | --- | --- | --- | --- | --- | --- | --- | --- |
|  |  | **Cases (%)** | **Cases (%)** | **Crude** | **Adjusted** | **Fully adjusted** | **Cases (%)** | | **Crude** | **Adjusted** | **Fully adjusted** | **Cases (%)** | | **Crude** | **Adjusted** | **Fully adjusted** |  |
| **Psychotropic medication during pregnancy (yes)** | **Preterm birth (<37 weeks)** | 1,308 (7‧4%) | 530 (9‧7%) | **1·33 (1·20-1·49)** | **1·29 (1·15-1·44)** | **1·22 (1·09-1·37)** | 379 (8‧7%) | | **1·19 (1·06-1·34)** | **1·16 (1·03-1·31)** | 1·11 (0·98-1·26) | 431 (10‧0%) | | **1·38 (1·23-1·55)** | **1·32 (1·17-1·49)** | **1·21 (1·07-1·37)** |  |
|  | **Low birthweight (<2500 g)** | 944 (5‧4%) | 351 (6‧4%) | **1·21 (1·06-1·37)** | 1·11 (0·98-1·27) | 1·03 (0·90-1·17) | 270 (6‧2%) | | **1·17 (1·02-1·34)** | 1·11 (0·97-1·29) | 1·05 (0·91-1·21) | 296 (6‧8%) | | **1·30 (1·13-1·49)** | **1·17 (1·01-1·36)** | 1·01 (0·87-1·18) |  |
|  | **Small for gestational age** | 1,996 (11%) | 642 (12%) | 1·04 (0·94-1·14) | 0·98 (0·88-1·08) | 0·92 (0·84-1·02) | 563 (13%) | | **1·16 (1·05-1·29)** | 1·07 (0·97-1·19) | 1·04 (0·93-1·15) | 588 (14%) | | **1·23 (1·11-1·36)** | 1·07 (0·96-1·19) | 0·97 (0·87-1·08) |  |
|  | **Apgar score at 5 minutes (< 7)** | 228 (1‧3%) | 88 (1‧6%) | 1·24 (0·97-1·60) | 1·09 (0·85-1·41) | 1·07 (0·83-1·38) | 66 (1‧5%) | | 1·17 (0·89-1·55) | 1·18 (0·89-1·57) | 1·16 (0·87-1·54) | 80 (1‧8%) | | **1·44 (1·11-1·86)** | 1·28 (0·98-1·66) | 1·23 (0·94-1·6) |  |
|  | **Caesarean delivery** | 4,332 (25%) | 1,486 (27%) | **1·14 (1·06-1·23)** | 1·05 (0·97-1·13) | 1·03 (0·95-1·11) | 1,045 (24%) | | 0·97 (0·90-1·05) | 1·03 (0·95-1·12) | 1‧00 (0·92-1·09) | 1,116 (26%) | | 1·07 (0·98-1·16) | 1·03 (0·95-1·12) | 0·97 (0·89-1·06) |  |
|  | **Neonatal death** | 40 (0‧2%) | 14 (0‧3%) | 1·12 (0·61-2·07) | 1·21 (0·64-2·28) | 1·13 (0·60-2·12) | 16 (0‧4%) | | 1·62 (0·91-2·90) | 1·61 (0·90-2·86) | 1·54 (0·86-2·75) | 6 (0‧1%) | | 0·61 (0·26-1·44) | 0·62 (0·26-1·49) | 0·56 (0·23-1·36) |  |
| **Psychotropic medication during pregnancy (no)** | **Preterm birth (<37 weeks)** | 49,271 (4‧7%) | 2,507 (5‧8%) | **1·24 (1·19-1·30)** | **1·19 (1·14-1·25)** | 1·04 (0·99-1·09) | 756 (6‧5%) | | **1·41 (1·31-1·52)** | **1·23 (1·14-1·33)** | 1·03 (0·95-1·11) | 506 (7‧7%) | | **1·67 (1·53-1·84)** | **1·44 (1·31-1·58)** | **1·15 (1·04-1·26)** |  |
|  | **Low birthweight (<2500 g)** | 34,171 (3‧3%) | 1,885 (4‧4%) | **1·34 (1·28-1·41)** | **1·22 (1·16-1·29)** | 1·01 (0·96-1·07) | 569 (4‧9%) | | **1·53 (1·40-1·66)** | **1·22 (1·12-1·33)** | 0·96 (0·88-1·05) | 401 (6‧1%) | | **1·91 (1·72-2·12)** | **1·46 (1·32-1·62)** | 1·08 (0·97-1·21) |  |
|  | **Small for gestational age** | 99,619 (9‧6%) | 4,774 (11%) | **1·17 (1·14-1·21)** | **1·08 (1·04-1·11)** | 0·98 (0·95-1·02) | 1,431 (12%) | | **1·33 (1·26-1·41)** | 1·06 (1‧00-1·12) | 0·95 (0·89-1‧00) | 885 (13%) | | **1·47 (1·36-1·58)** | **1·11 (1·03-1·20)** | 0·96 (0·89-1·04) |  |
|  | **Apgar score at 5 minutes (< 7)** | 7,035 (0‧7%) | 290 (0‧7%) | 0·99 (0·88-1·12) | 0·97 (0·86-1·10) | 0·92 (0·82-1·04) | 84 (0·7%) | | 1·08 (0·87-1·33) | 0·99 (0·80-1·24) | 0·93 (0·74-1·15) | 61 (0‧9%) | | **1·37 (1·07-1·77)** | 1·24 (0·96-1·59) | 1·13 (0·87-1·45) |  |
|  | **Caesarean delivery** | 187,282 (18%) | 9,128 (21%) | **1·22 (1·19-1·26)** | **1·14 (1·10-1·17)** | **1·05 (1·02-1·08)** | 2,246 (19%) | | **1·10 (1·05-1·15)** | **1·15 (1·10-1·21)** | 1·04 (0·99-1·09) | 1,389 (21%) | | **1·22 (1·14-1·30)** | **1·17 (1·09-1·24)** | 1·01 (0·95-1·08) |  |
|  | **Neonatal death** | 1,926 (0‧2%) | 77 (0‧2%) | 0·96 (0·77-1·21) | 0·97 (0·77-1·23) | 0·85 (0·67-1·07) | 24 (0·2%) | | 1·12 (0·75-1·68) | 0·98 (0·65-1·46) | 0·80 (0·54-1·21) | 15 (0‧2%) | | 1·23 (0·74-2·05) | 1·11 (0·67-1·86) | 0·88 (0·53-1·48) |  |

**Crude:** No adjustments.

**Adjusted:** Adjusted for maternal age at delivery; parity; marital status; highest education; calendar year of delivery.

**Fully adjusted:** Adjusted for maternal age at delivery; parity; marital status; highest education; calendar year of delivery; number of non-psychiatric hospital visits during pregnancy; smoking during pregnancy, pregnancy complications.

# Supplementary Table 7. Sensitivity analysis: Crude, adjusted, and fully adjusted odds ratios (ORs) with 95% confidence intervals (CIs) for the associations between maternal mental disorders and birth outcomes, including maternal low birthweight as a confounder.

The population for this analysis consists of 602,880 children born to 329,948 mothers, as the population is restricted to those for whom information on maternal low birthweight is available.

|  | **No mental disorders**  **(N = 550,137)** | **Past only mental disorders**  **(N = 34,703)** | | | | | | **Recent only mental disorders**  **(N = 10,070)** | | | | | | **Persistent mental disorders**  **(N = 7,970)** | | | | | |
| --- | --- | --- | --- | --- | --- | --- | --- | --- | --- | --- | --- | --- | --- | --- | --- | --- | --- | --- | --- |
| **Outcome** | **Cases (%)** | **Cases (%)** | **Crude** | **Adj·** | **Adj· + LBW** | **Fully adj·** | **Fully adj· + LBW** | **Cases (%)** | **Crude** | **Adj·** | **Adj· + LBW** | **Fully adj·** | **Fully adj· + LBW** | **Cases (%)** | **Crude** | **Adj·** | **Adj· + LBW** | **Fully adj·** | **Fully adj· + LBW** |
| **Preterm birth (<37 weeks)** | 26,836 (4‧9%) | 2,114 (6‧1%) | **1·26 (1·20-1·33)** | **1·24 (1·18-1·30)** | **1·23 (1·17-1·29)** | **1·10 (1·05-1·16)** | **1·10 (1·04-1·15)** | 708 (7‧0%) | **1·47 (1·36-1·59)** | **1·28 (1·18-1·38)** | **1·27 (1·17-1·37)** | **1·10 (1·01-1·19)** | **1·09 (1·01-1·18)** | 642 (8‧1%) | **1·71 (1·57-1·86)** | **1·50 (1·38-1·63)** | **1·49 (1·37-1·62)** | **1·22 (1·12-1·34)** | **1·22 (1·12-1·33)** |
| **Low birthweight (<2500 g)** | 17,994 (3‧3%) | 1,537 (4‧4%) | **1·37 (1·29-1·45)** | **1·26 (1·19-1·34)** | **1·25 (1·18-1·33)** | **1·07 (1·01-1·14)** | 1·06 (1‧00-1·13) | 527 (5‧2%) | **1·63 (1·49-1·79)** | **1·28 (1·17-1·40)** | **1·26 (1·15-1·38)** | 1·03 (0·94-1·13) | 1·02 (0·93-1·12) | 474 (5‧9%) | **1·87 (1·70-2·06)** | **1·45 (1·31-1·60)** | **1·43 (1·29-1·58)** | 1·09 (0·99-1·21) | 1·08 (0·98-1·20) |
| **Small for gestational age** | 51,512 (9‧4%) | 3,729 (11%) | **1·17 (1·12-1·21)** | **1·09 (1·05-1·13)** | **1·08 (1·04-1·12)** | 1·01 (0·97-1·05) | 1‧00 (0·96-1·04) | 1,273 (13%) | **1·40 (1·32-1·49)** | **1·12 (1·05-1·19)** | **1·11 (1·04-1·18)** | 1‧00 (0·94-1·06) | 0·99 (0·93-1·06) | 1,040 (13%) | **1·45 (1·36-1·55)** | **1·14 (1·06-1·22)** | **1·13 (1·05-1·21)** | 0·99 (0·93-1·07) | 0·99 (0·92-1·06) |
| **Apgar score at 5 minutes (< 7)** | 3,675 (0‧7%) | 272 (0‧8%) | **1·17 (1·04-1·33)** | 1·13 (0·99-1·28) | 1·12 (0·99-1·28) | 1·07 (0·94-1·22) | 1·07 (0·94-1·21) | 92 (0‧9%) | **1·37 (1·11-1·69)** | 1·23 (0·99-1·51) | 1·22 (0·99-1·51) | 1·14 (0·93-1·41) | 1·14 (0·92-1·41) | 107 (1‧3%) | **2·02 (1·67-2·45)** | **1·76 (1·45-2·15)** | **1·76 (1·44-2·14)** | **1·61 (1·32-1·96)** | **1·60 (1·32-1·96)** |
| **Caesarean delivery** | 99,385 (18%) | 7,244 (21%) | **1·20 (1·16-1·24)** | **1·14 (1·11-1·18)** | **1·14 (1·10-1·18)** | **1·06 (1·03-1·10)** | **1·06 (1·03-1·10)** | 1,993 (20%) | **1·12 (1·06-1·18)** | **1·19 (1·13-1·25)** | **1·18 (1·12-1·24)** | **1·08 (1·02-1·14)** | **1·08 (1·02-1·13)** | 1,744 (22%) | **1·27 (1·20-1·35)** | **1·23 (1·16-1·30)** | **1·22 (1·15-1·30)** | **1·08 (1·02-1·14)** | **1·08 (1·01-1·14)** |
| **Neonatal death** | 896 (0‧2%) | 59 (0‧2%) | 1·04 (0·80-1·36) | 0·99 (0·76-1·30) | 0·99 (0·76-1·30) | 0·87 (0·66-1·13) | 0·86 (0·66-1·13) | 26 (0‧3%) | **1·59 (1·07-2·34)** | 1·25 (0·84-1·85) | 1·24 (0·84-1·85) | 1·03 (0·69-1·53) | 1·03 (0·69-1·53) | 11 (0‧1%) | 0·85 (0·47-1·54) | 0·70 (0·38-1·27) | 0·69 (0·38-1·26) | 0·55 (0·30-1‧00) | 0·55 (0·30-1‧00) |

**Crude:** No adjustments

**Adj:** Adjusted for maternal age at delivery; parity; marital status; highest education; calendar year of delivery

**Adj + LBW:** Adjusted for maternal age at delivery; parity; marital status; highest education; calendar year of delivery; maternal low birthweight.

**Fully adj:** Adjusted for maternal age at delivery; parity; marital status; highest education; calendar year of delivery; number of non-psychiatric hospital visits during pregnancy; smoking during pregnancy, pregnancy complications

**Fully adj + LBW:** Adjusted for maternal age at delivery; parity; marital status; highest education; calendar year of delivery; number of non-psychiatric hospital visits during pregnancy; smoking during pregnancy, pregnancy complications; maternal low birthweight

# Supplementary Figure 3. Crude, adjusted, and fully adjusted odds ratios (ORs) with 95% confidence intervals (CIs) for the associations between maternal mental disorders and birth outcomes in the main analysis (a) compared with sensitivity analyses relating to firstborn children only (b) and maternal low birthweight (LBW) as a potential confounder (c).


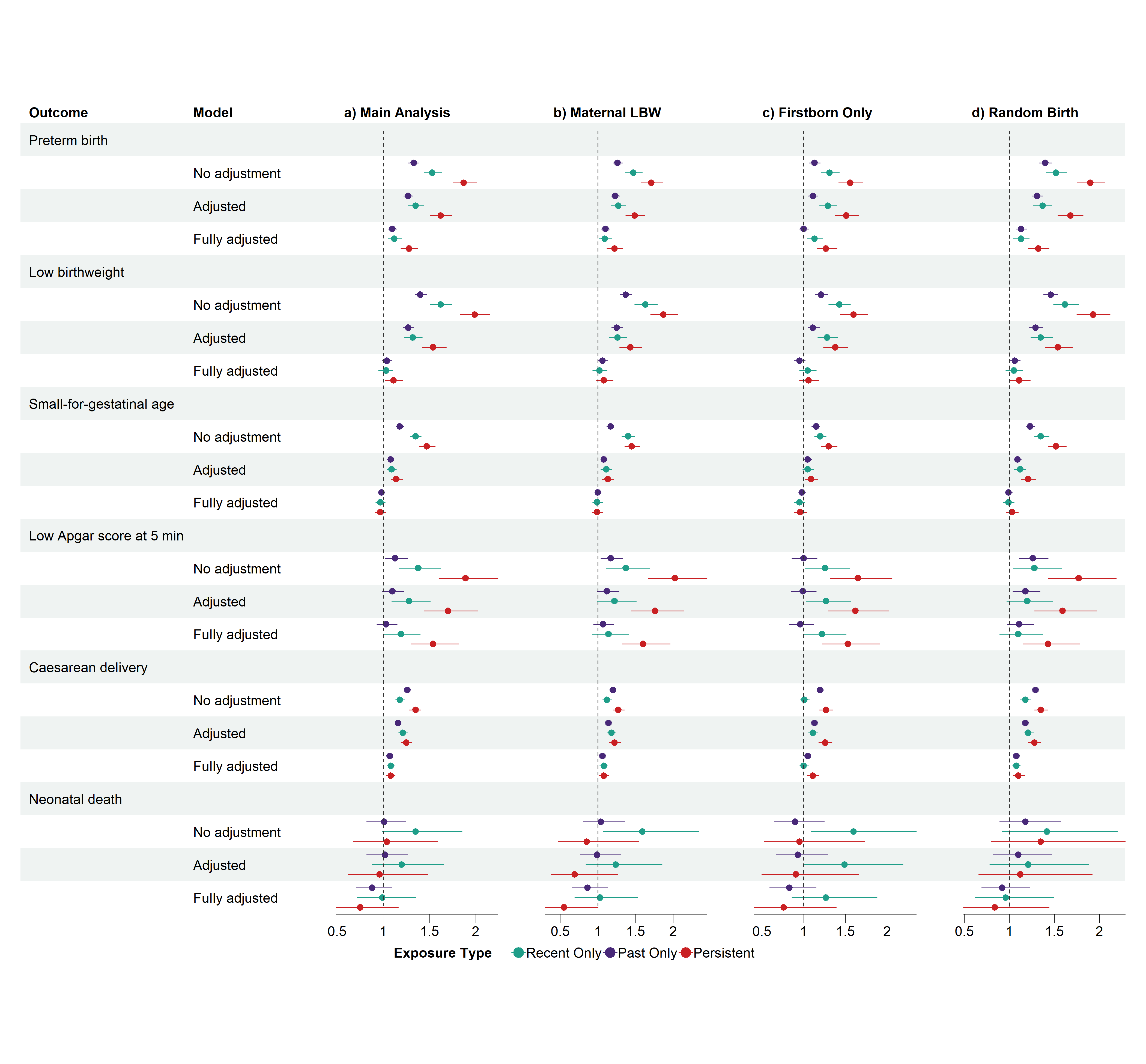


# Supplementary Table 8. Sensitivity analysis: Crude, adjusted, and fully adjusted odds ratios (ORs) with 95% confidence intervals (CIs) for the associations between maternal mental disorders and birth outcomes, among firstborn children only.

The population for this analysis consists of 494,354 children, as the population was restricted to firstborn children only.

| **Outcome** | **No mental disorders**  **(N = 461,215)** | **Past only mental disorders**  **(N = 20,762)** | | | | **Recent only mental disorders**  **(N = 8,345)** | | | | **Persistent mental disorders**  **(N = 5,691)** | | | |
| --- | --- | --- | --- | --- | --- | --- | --- | --- | --- | --- | --- | --- | --- |
|  | **Cases (%)** | **Cases (%)** | **Crude** | **Adjusted** | **Fully adjusted** | **Cases (%)** | **Crude** | **Adjusted** | **Fully adjusted** | **Cases (%)** | **Crude** | **Adjusted** | **Fully adjusted** |
| **Preterm birth (<37 weeks)** | 27,710 (6·0%) | 1,399 (6·8%) | **1·13 (1·07-1·20)** | **1·11 (1·05-1·17)** | 1‧00 (0·95-1·06) | 645 (7·8%) | **1·31 (1·21-1·43)** | **1·29 (1·19-1·40)** | **1·13 (1·04-1·23)** | 515 (9·1%) | **1·56 (1·42-1·71)** | **1·51 (1·38-1·66)** | **1·27 (1·16-1·40)** |
| **Low birthweight (<2500 g)** | 20,215 (4·4%) | 1,090 (5·3%) | **1·21 (1·14-1·29)** | **1·11 (1·05-1·19)** | 0·95 (0·89-1·02) | 511 (6·2%) | **1·43 (1·30-1·56)** | **1·28 (1·17-1·41)** | 1·05 (0·95-1·15) | 388 (6·9%) | **1·60 (1·44-1·77)** | **1·38 (1·24-1·53)** | 1·06 (0·95-1·18) |
| **Small for gestational age** | 58,910 (13%) | 2,985 (14%) | **1·15 (1·10-1·19)** | **1·05 (1·01-1·10)** | 0·98 (0·94-1·02) | 1,243 (15%) | **1·20 (1·13-1·27)** | 1·05 (0·99-1·12) | 0·95 (0·89-1·01) | 908 (16%) | **1·30 (1·21-1·40)** | **1·09 (1·02-1·17)** | 0·96 (0·89-1·04) |
| **Apgar score at 5 minutes (< 7)** | 4,058 (0·9%) | 183 (0·9%) | 1‧00 (0·86-1·16) | 0·99 (0·85-1·15) | 0·96 (0·83-1·12) | 92 (1·1%) | **1·26 (1·02-1·55)** | **1·27 (1·03-1·57)** | 1·22 (0·99-1·51) | 82 (1·4%) | **1·65 (1·32-2·06)** | **1·62 (1·29-2·02)** | **1·53 (1·22-1·91)** |
| **Caesarean delivery** | 91,877 (20%) | 4,776 (23%) | **1·20 (1·16-1·24)** | **1·13 (1·09-1·17)** | **1·05 (1·01-1·09)** | 1,673 (20%) | 1·01 (0·96-1·07) | **1·11 (1·05-1·17)** | 1‧00 (0·95-1·06) | 1,364 (24%) | **1·27 (1·19-1·35)** | **1·26 (1·18-1·34)** | **1·11 (1·04-1·18)** |
| **Neonatal death** | 937 (0·2%) | 38 (0·2%) | 0·90 (0·65-1·25) | 0·93 (0·67-1·29) | 0·83 (0·59-1·15) | 27 (0·3%) | **1·60 (1·09-2·35)** | **1·49 (1·01-2·19)** | 1·27 (0·86-1·88) | 11 (0·2%) | 0·95 (0·53-1·73) | 0·91 (0·50-1·66) | 0·76 (0·41-1·39) |

**Crude:** No adjustments.

**Adjusted:** Adjusted for maternal age at delivery; marital status; highest education; calendar year of delivery.

**Fully adjusted:** Adjusted for maternal age at delivery; marital status; highest education; calendar year of delivery; number of non-psychiatric hospital visits during pregnancy; smoking during pregnancy, pregnancy complications.

# Supplementary Table 9. Sensitivity analysis: Crude, adjusted, and fully adjusted odds ratios (ORs) with 95% confidence intervals (CIs) for the associations between maternal mental disorders and birth outcomes, with the population restricted to one random birth per mother.

The population for this analysis consists of 663,345 children, as the population was restricted to one random birth per mother.

| **Outcome** | **No mental disorders**  **(N = 617,675)** | **Past only mental disorders**  **(N = 28,717)** | | | | **Recent only mental disorders**  **(N = 9,897)** | | | | | **Persistent mental disorders**  **(N = 7,056)** | | | |
| --- | --- | --- | --- | --- | --- | --- | --- | --- | --- | --- | --- | --- | --- | --- |
|  | **Cases (%)** | **Cases (%)** | **Crude** | **Adjusted** | **Fully adjusted** | **Cases (%)** | **Crude** | **Adjusted** | **Fully adjusted** | **Cases (%)** | | **Crude** | **Adjusted** | **Fully adjusted** |
| **Preterm birth (<37 weeks)** | 31,197 (5‧1%) | 1,919 (6‧7%) | **1·40 (1·33-1·47)** | **1·31 (1·25-1·37)** | **1·13 (1·08-1·19)** | 756 (7‧6%) | **1·52 (1·41-1·64)** | **1·37 (1·26-1·47)** | **1·13 (1·04-1·22)** | 631 (8‧9%) | | **1·90 (1·75-2·06)** | **1·68 (1·54-1·82)** | **1·32 (1·21-1·44)** |
| **Low birthweight (<2500 g)** | 22,357 (3‧6%) | 1,431 (5‧0%) | **1·46 (1·38-1·54)** | **1·29 (1·22-1·37)** | 1·06 (1‧00-1·12) | 572 (5‧8%) | **1·62 (1·49-1·77)** | **1·35 (1·24-1·48)** | 1·05 (0·96-1·15) | 464 (6‧6%) | | **1·93 (1·75-2·12)** | **1·54 (1·40-1·70)** | 1·11 (1‧00-1·23) |
| **Small for gestational age** | 62,892 (10%) | 3,465 (12%) | **1·23 (1·19-1·28)** | **1·09 (1·05-1·13)** | 0·99 (0·96-1·03) | 1,314 (13%) | **1·35 (1·28-1·44)** | **1·12 (1·05-1·18)** | 0·99 (0·93-1·05) | 1,015 (14%) | | **1·52 (1·43-1·63)** | **1·21 (1·13-1·29)** | 1·03 (0·96-1·10) |
| **Apgar score at 5 minutes (< 7)** | 4,334 (0‧7%) | 220 (0‧8%) | **1·26 (1·11-1·43)** | **1·18 (1·04-1·34)** | 1·11 (0·98-1·27) | 100 (1‧0%) | **1·28 (1·04-1·58)** | 1·20 (0·97-1·48) | 1·10 (0·89-1·37) | 95 (1‧3%) | | **1·77 (1·43-2·19)** | **1·59 (1·28-1·97)** | **1·43 (1·15-1·78)** |
| **Caesarean delivery** | 116,719 (19%) | 6,584 (23%) | **1·29 (1·26-1·33)** | **1·18 (1·14-1·21)** | **1·08 (1·05-1·11)** | 2,114 (21%) | **1·18 (1·12-1·24)** | **1·21 (1·16-1·27)** | **1·08 (1·03-1·13)** | 1,678 (24%) | | **1·35 (1·28-1·43)** | **1·28 (1·21-1·35)** | **1·10 (1·04-1·17)** |
| **Neonatal death** | 920 (0‧1%) | 35 (0‧1%) | 1·18 (0·89-1·57) | 1·10 (0·82-1·47) | 0·92 (0·69-1·23) | 23 (0‧2%) | 1·42 (0·92-2·20) | 1·21 (0·78-1·88) | 0·96 (0·62-1·49) | 11 (0‧2%) | | 1·35 (0·80-2·29) | 1·12 (0·66-1·92) | 0·84 (0·49-1·44) |

**Crude:** No adjustments.

**Adjusted:** Adjusted for maternal age at delivery; marital status; highest education; calendar year of delivery.

**Fully adjusted:** Adjusted for maternal age at delivery; marital status; highest education; calendar year of delivery; number of non-psychiatric hospital visits during pregnancy; smoking during pregnancy; pregnancy complications.

# STROBE Statement—checklist of items that should be included in reports of observational studies

|  | Item No. | Recommendation | Page  No. | | Relevant text from manuscript |
| --- | --- | --- | --- | --- | --- |
| **Title and abstract** | 1 | (*a*) Indicate the study’s design with a commonly used term in the title or the abstract | 1 | | Maternal Mental Disorders and Neonatal Outcomes: a Danish Population-based Cohort Study |
|  |  | (*b*) Provide in the abstract an informative and balanced summary of what was done and what was found | 2 | | Methods: We included 1,132,757 liveborn singletons born between 1997 and 2015 in Denmark. We compared children of mothers with a past (>2 years prior to conception; n=48,646), recent (2 years prior to conception and during pregnancy; n=15,899) or persistent (both past and recent; n=10,905) diagnosis of any mental disorder, with children of mothers with no mental disorder diagnosis before the index delivery (n=1,057,307). We also considered different types of mental disorders. We calculated odds ratios (ORs) and 95% confidence intervals (CIs) of low birthweight, preterm birth, small for gestational age (SGA), low Apgar score, Caesarean delivery, and neonatal death.  Results: ORs for children exposed to past, recent and persistent maternal mental disorders suggested an increased risk for almost all adverse neonatal outcomes. Estimates were highest for children in the “persistent” group for all outcomes, with the exception of the association between persistent maternal mental disorders and neonatal death (OR 0·96, 0·62-1·48). |
| Introduction | | | |  | |
| Background/rationale | 2 | Explain the scientific background and rationale for the investigation being reported | 3 | | A broad study examining all mental disorder types would allow for comparison and provide a more complete understanding of the associations between maternal mental disorders and adverse neonatal outcomes. |
| Objectives | 3 | State specific objectives, including any prespecified hypotheses | 3 | | In this study, we used Danish nationwide registers to investigate adverse neonatal outcomes among offspring whose mothers had been diagnosed with a mental disorder. Compared with children of mothers with no mental disorder diagnosis, we considered children of mothers with i) a past mental disorder diagnosis (more than 2 years prior to conception), ii) a recent mental disorder diagnosis (2 years prior to conception and during pregnancy) and iii) a persistent mental disorder diagnosis (both past and recent diagnoses). |
| Methods | | | |  | |
| Study design | 4 | Present key elements of study design early in the paper | 4 | | We carried out a population-based cohort study using Danish registers. |
| Setting | 5 | Describe the setting, locations, and relevant dates, including periods of recruitment, exposure, follow-up, and data collection | 4 | | We identified all liveborn singletons born between 1997 and 2015 (n=1,159,624) from the Danish Medical Birth Register (MBR). All residents in Denmark are assigned a unique identification number in the Danish Civil Registration System, which allows linkage of data between different registers. |
| Participants | 6 | (*a*) *Cohort study*—Give the eligibility criteria, and the sources and methods of selection of participants. Describe methods of follow-up  *Case-control study*—Give the eligibility criteria, and the sources and methods of case ascertainment and control selection. Give the rationale for the choice of cases and controls  *Cross-sectional study*—Give the eligibility criteria, and the sources and methods of selection of participants | 4 | | We identified all liveborn singletons born between 1997 and 2015 (n=1,159,624) from the Danish Medical Birth Register (MBR). All residents in Denmark are assigned a unique identification number in the Danish Civil Registration System, which allows linkage of data between different registers.  We selected seven outcomes relating to labor, delivery, and neonatal complications based on research highlighting these outcomes as associated with maternal mental disorders.1, 3 Most outcomes were ascertained using the MBR: preterm birth (gestational age <37 weeks), low birthweight (birthweight <2500g), SGA (i.e., birthweight <10 percentile for sex and gestational age), low 5-minute Apgar score (<7), and Caesarean delivery (surgery codes KMCA10-KMCA12). Neonatal deaths (death from any cause within 28 days of birth) were identified in the Danish Civil Registration System. |
|  |  | (*b*) *Cohort study*—For matched studies, give matching criteria and number of exposed and unexposed  *Case-control study*—For matched studies, give matching criteria and the number of controls per case | N/A | |  |
| Variables | 7 | Clearly define all outcomes, exposures, predictors, potential confounders, and effect modifiers. Give diagnostic criteria, if applicable | 4-5 | | Sections *Exposure: Maternal mental disorder diagnosis*, *Outcomes: Neonatal outcomes* and *Potential confounders* within the Methods section |
| Data sources/ measurement | 8* | For each variable of interest, give sources of data and details of methods of assessment (measurement). Describe comparability of assessment methods if there is more than one group | 4-5 | | Sections *Exposure: Maternal mental disorder diagnosis*, *Outcomes: Neonatal outcomes* and *Potential confounders* within the Methods section |
| Bias | 9 | Describe any efforts to address potential sources of bias | 6-7 | | Sections *Additional analyses* within the Methods section |
| Study size | 10 | Explain how the study size was arrived at | Figure 1, 7 | | We identified all liveborn singletons born between 1997 and 2015 (n=1,159,624) from the Danish Medical Birth Register (MBR). All residents in Denmark are assigned a unique identification number in the Danish Civil Registration System, which allows linkage of data between different registers.  We excluded 23,676 children with missing or unlikely information on outcomes (gestational age <154 or >315 days, or missing; birthweight <300 or >6400 grams, or missing; missing Apgar score) and 3,191 children with chromosomal abnormalities (ICD-10 [International Classification of Diseases, 10th revision] codes Q90–Q99) identified from the Danish National Patient Register. This resulted in a sample of 1,132,757 singletons born to 663,345 mothers (Figure 1). |
| Quantitative variables | 11 | Explain how quantitative variables were handled in the analyses. If applicable, describe which groupings were chosen and why | 6 | Sections *Exposure: Maternal mental disorder diagnosis* and *Outcomes: Neonatal outcomes* within the Methods section | |
| Statistical methods | 12 | (*a*) Describe all statistical methods, including those used to control for confounding | 6 | Sections *Statistical analysis* within the Methods section | |
|  |  | (*b*) Describe any methods used to examine subgroups and interactions | 6-7 | Sections *Additional analyses* within the Methods section | |
|  |  | (*c*) Explain how missing data were addressed | 6 | For marital status, smoking, and education covariates, 0·5%, 3·3%, and 3·4% of values were missing, respectively; we applied multivariate logistic regression imputation by chained equations with 20 imputations to impute missing values. | |
|  |  | (*d*) *Cohort study*—If applicable, explain how loss to follow-up was addressed  *Case-control study*—If applicable, explain how matching of cases and controls was addressed  *Cross-sectional study*—If applicable, describe analytical methods taking account of sampling strategy | N/A as outcomes were measured at birth |  | |
|  |  | (*e*) Describe any sensitivity analyses | 6-7 | Sections *Additional analyses* within the Methods section | |
| Results | | | | | |
| Participants | 13* | (a) Report numbers of individuals at each stage of study—eg numbers potentially eligible, examined for eligibility, confirmed eligible, included in the study, completing follow-up, and analysed | Figure 1, 7 | We identified all liveborn singletons born between 1997 and 2015 (n=1,159,624) from the Danish Medical Birth Register (MBR). All residents in Denmark are assigned a unique identification number in the Danish Civil Registration System, which allows linkage of data between different registers.  We excluded 23,676 children with missing or unlikely information on outcomes (gestational age <154 or >315 days, or missing; birthweight <300 or >6400 grams, or missing; missing Apgar score) and 3,191 children with chromosomal abnormalities (ICD-10 [International Classification of Diseases, 10th revision] codes Q90–Q99) identified from the Danish National Patient Register. This resulted in a sample of 1,132,757 singletons born to 663,345 mothers (Figure 1). | |
|  |  | (b) Give reasons for non-participation at each stage | Figure 1, 7 | See above | |
|  |  | (c) Consider use of a flow diagram | Figure 1 |  | |
| Descriptive data | 14* | (a) Give characteristics of study participants (eg demographic, clinical, social) and information on exposures and potential confounders | Table 1 |  | |
|  |  | (b) Indicate number of participants with missing data for each variable of interest | 6 | For marital status, smoking, and education covariates, 0·5%, 3·3%, and 3·4% of values were missing, respectively; we applied multivariate logistic regression imputation by chained equations with 20 imputations to impute missing values. | |
|  |  | (c) *Cohort study*—Summarise follow-up time (eg, average and total amount) | N/A as outcomes were assessed at birth |  | |
| Outcome data | 15* | *Cohort study*—Report numbers of outcome events or summary measures over time | Table 2 |  | |
|  |  | *Case-control study—*Report numbers in each exposure category, or summary measures of exposure |  |  | |
|  |  | *Cross-sectional study—*Report numbers of outcome events or summary measures |  |  | |
| Main results | 16 | (*a*) Give unadjusted estimates and, if applicable, confounder-adjusted estimates and their precision (eg, 95% confidence interval). Make clear which confounders were adjusted for and why they were included | 5, Figure 2, Supplementary Table 2 | Sections *Potential confounders* within the Methods section | |
|  |  | (*b*) Report category boundaries when continuous variables were categorized | 5, Figure 2, Supplementary Table 2 | Sections *Potential confounders* within the Methods section | |
|  |  | (*c*) If relevant, consider translating estimates of relative risk into absolute risk for a meaningful time period |  |  | |

| Other analyses | 17 | Report other analyses done—eg analyses of subgroups and interactions, and sensitivity analyses | 6-7 | Sections *Additional analyses* within the Results section |
| --- | --- | --- | --- | --- |
| Discussion | | | | |
| Key results | 18 | Summarise key results with reference to study objectives | 10 | We found that the risk of preterm birth, low birthweight, SGA, low Apgar score, Caesarean delivery, and neonatal death were increased among children in any of the exposure groups: any past, recent or persistent maternal mental disorder diagnoses. For these outcomes, we observed higher point estimates among children whose mothers had a persistent diagnosis than among children whose mothers had a past or recent diagnosis. However, neonatal death was increased only among children whose mothers had a past or recent diagnosis only, not persistent diagnoses, which appears contradictory. |
| Limitations | 19 | Discuss limitations of the study, taking into account sources of potential bias or imprecision. Discuss both direction and magnitude of any potential bias | 12-13 | Section *Strengths and limitations* within the Discussion section |
| Interpretation | 20 | Give a cautious overall interpretation of results considering objectives, limitations, multiplicity of analyses, results from similar studies, and other relevant evidence | 13-14 | Section *Conclusions* within the Discussion section |
| Generalisability | 21 | Discuss the generalisability (external validity) of the study results | 13 | Finally, while our results support those reported in previous studies, the generalizability of our results outside of Denmark is unclear. |
| Other information | |  | | |
| Funding | 22 | Give the source of funding and the role of the funders for the present study and, if applicable, for the original study on which the present article is based | 14 | Section *Funding* |

*Give information separately for cases and controls in case-control studies and, if applicable, for exposed and unexposed groups in cohort and cross-sectional studies.

**Note:** An Explanation and Elaboration article discusses each checklist item and gives methodological background and published examples of transparent reporting. The STROBE checklist is best used in conjunction with this article (freely available on the Web sites of PLoS Medicine at http://www.plosmedicine.org/, Annals of Internal Medicine at http://www.annals.org/, and Epidemiology at http://www.epidem.com/). Information on the STROBE Initiative is available at www.strobe-statement.org.
